# Phosphorus Starvation- and Zinc Excess-Induced *Astragalus sinicus* AsZIP2 Zinc Transporter Is Suppressed by Arbuscular Mycorrhizal Symbiosis

**DOI:** 10.3390/jof7110892

**Published:** 2021-10-22

**Authors:** Xianan Xie, Xiaoning Fan, Hui Chen, Ming Tang

**Affiliations:** State Key Laboratory of Conservation and Utilization of Subtropical Agro-Bioresources, Guangdong Laboratory for Lingnan Modern Agriculture, Guangdong Key Laboratory for Innovative Development and Utilization of Forest Plant Germplasm, College of Forestry and Landscape Architecture, South China Agricultural University, Guangzhou 510642, China; 30004537@scau.edu.cn (X.X.); fanxiaoning@stu.scau.edu.cn (X.F.); chenhui@scau.edu.cn (H.C.)

**Keywords:** Zinc, phosphate, arbuscular mycorrhizal fungi, *Astragalus sinicus*, *ZIP* gene family, AsZIP2 transporter, crosstalk between Pi-Zn

## Abstract

Zinc (Zn) is one of the most essential micronutrients for plant growth and metabolism, but Zn excess can impair many basic metabolic processes in plant cells. In agriculture, crops often experience low phosphate (Pi) and high Zn double nutrient stresses because of inordinate agro-industrial activities, while the dual benefit of arbuscular mycorrhizal (AM) fungi protects plants from experiencing both deficient and toxic nutrient stresses. Although crosstalk between Pi and Zn nutrients in plants have been extensively studied at the physiological level, the molecular basis of how Pi starvation triggers Zn over-accumulation in plants and how AM plants coordinately modulate the Pi and Zn nutrient homeostasis remains to be elucidated. Here, we report that a novel *AsZIP2* gene, a Chinese milk vetch (*Astragalus sinicus*) member of the *ZIP* gene family, participates in the interaction between Pi and Zn nutrient homeostasis in plants. Phylogenetic analysis revealed that this AsZIP2 protein was closely related to the orthologous Medicago MtZIP2 and Arabidopsis AtZIP2 transporters. Gene expression analysis indicated that *AsZIP2* was highly induced in roots by Pi starvation or Zn excess yet attenuated by arbuscular mycorrhization in a Pi-dependent manner. Subcellular localization and heterologous expression experiments further showed that *AsZIP2* encoded a functional plasma membrane-localized transporter that mediated Zn uptake in yeast. Moreover, overexpression of *AsZIP2* in *A. sinicus* resulted in the over-accumulation of Zn concentration in roots at low Pi or excessive Zn concentrations, whereas *AsZIP2* silencing lines displayed an even more reduced Zn concentration than control lines under such conditions. Our results reveal that the AsZIP2 transporter functioned in Zn over-accumulation in roots during Pi starvation or high Zn supply but was repressed by AM symbiosis in a Pi-dependent manner. These findings also provide new insights into the *AsZIP2* gene acting in the regulation of Zn homeostasis in mycorrhizal plants through Pi signal.

## 1. Introduction

In agriculture, the dynamics in availability of soil nutrients affect crop growth and yield and thereby the optimal fertilization of micro-nutrients in a field is important for healthy and sustainable agriculture production [1]. Zinc (Zn) is one of the most essential micronutrients for plant growth and metabolism [2] and is also a “double-edged sword” due to its heavy metal properties. Both deficiency and excess in this heavy metal Zn can impair many basic physiological and metabolic processes in plant cells [3,4]. On one hand, Zn^2+^ deficiency often occurs in high pH calcareous soils and high phosphorus (P) fertilized soils [2], and some forms of Zn derivates such as zinc oxide, calcium zincate, zinc silicate and zinc sulfide in soil are relatively inaccessible to plants due to their low solubility and relative immobilization [5,6]. However, higher plants have evolved a complex array of adaptive strategies to cope with the low availability of soil Zn, including an enhanced efficiency of Zn uptake from the soil [7], an increase in the export of stored Zn from the vacuoles, Zn redistribution from the senescent to young expanding organs [8], bioweathering of zinc silicate and sulfide by saprotrophic fungi [6], and development of mutual symbioses with soil microbes [9,10,11,12]. On the other hand, when present in excess, soil Zn^2+^, ZnO nanoparticles (NPs) and their derivatives such as ZnO NPs-derived Zn^2+^ can cause both toxicity and Zn over-accumulation in plants [13,14,15], plants must also develop a variety of controlled mechanisms to combat toxicity from excessive Zn. These include the efflux of excessive Zn out of the cell or regulating Zn uptake at the plasma membrane, sequestration of excessive Zn in the vacuoles [16,17,18,19], and the more specialized alleviation mechanism of arbuscular mycorrhizas (AM) in plant roots [12,20]. Recent studies have revealed that plants depend on the regulation of Zn transporters, including Zinc-regulated/Iron-regulated transporter-like Proteins (ZIP) family transporters [21,22,23], natural resistance-associated macrophage proteins (NRAMPs) [24], and the cation diffusion facilitator (CDF) family efflux transporters [25,26,27] to control intracellular Zn homeostasis. Therefore, these heavy metal transporters play an irreplaceable role in the regulation of Zn acquisition, sequestration, translocation, and redistribution in plants at the cellular level [28,29,30,31].

In plants, Zn is predominantly acquired at the root periphery by free ions (divalent Zn^2+^) [32,33], and subsequent Zn^2+^ transport into the cortical cells takes place through the symplastic or apoplastic pathway [33]. The different types of Zn transporters as described above are required to import Zn^2+^ into the cells and export them from intracellular compartments such as the vacuoles. These Zn ion transporters include both high-affinity and low-affinity membrane transporter systems [7,33]. The high-affinity transporter system is mainly activated at the root surface during Zn deficiency in soil [7]; these transporters, belonging to the ZIP family, are considered to be Zn uptake transporters, and import Zn^2+^ ions across the plasma membranes into the cytoplasm [1,34,35]. After that Zn^2+^ ions pass the endodermis, then the two heavy-metal ATPase 2 (HMA2) and HMA4 plasma membrane transporters belonging to the P_1B_-type ATPase family play vital roles, participating in xylem loading of Zn from the xylem parenchymatous cells [36,37]. A more recent study has proposed that the ZIP family transporters are mainly involved in uptake, transport, and distribution of Zn in the various plant tissues [1]. Therefore, a better understanding of the expression profiles, tissue localization, and biological functions of ZIP family transporters in crop species is essential.

There are fifteen and sixteen members of the ZIP gene family identified in the genomes of *Arabidopsis thaliana* [17] and rice (*Oryza sativa*) [38,39], respectively. Nevertheless, most of the ZIP transporters in crops are poorly understood, and few have been functionally characterized in plants [21,22,23,40,41,42]. Most of the plant ZIP family genes are induced in roots and shoots during Zn starvation [32,39,40,41,43,44], suggesting their primary role in adapting to Zn-deficiency stress. By contrast, it has been reported that *MtZIP2* from *Medicago truncatula* and *AtZIP6* from *A. thaliana* are up-regulated in the roots exposed to Zn excess [45,46]; moreover, recent studies have shown that *NtZIP1-like* and *NtZIP11* from tobacco plants are specifically up-regulated in the leaves by toxic Zn levels [21,47]. These findings indicate that these two ZIP genes may be involved in the accumulation of Zn in the leave cells of tobacco plants exposed to high Zn concentrations. Therefore, there may exist some ZIP transporters that possibly participate in the over-accumulation of Zn in plants exposed to Zn excess, although they have not been functionally characterized. However, it is not known whether new Zn transporters of the ZIP family in dicotyledonous species are able to result in an enhanced Zn accumulation in roots when their expression is highly increased during Zn excess.

In nature ecosystems, higher plants, as sessile organisms, are faced with multiple nutrient stresses that often occur simultaneously, such as those associated with Pi and Zn. Crosstalk between Pi and Zn nutrients in plants have long been recognized and are relatively well understood at the physiological level [48,49,50,51,52,53]. The antagonistic interaction between Pi and Zn nutrients in different plant species can be summarized as follows: Pi starvation leads to the over-accumulation of Zn concentration in plants, and inversely, Zn deficiency results in increased Pi concentration in plants. However, little information is available on the molecular bases of these Pi and Zn nutrient interactions in plants [51,54,55,56]. Recently, it has been proposed that Phosphate-deficiency Response 1 (PHR1) factor, members of the Phosphate 1 (PHO1, PHO1; H1, and PHO1; H3) family, and HMA2 and HMA4 transporters participate in the coordination between Pi and Zn homeostasis in *Arabidopsis* [50,56,57]. More recently, a study has shown that high Zn levels induce Pi starvation by inhibiting root-to-shoot translocation and distribution of Pi into new leaves of rice plants by down-regulating Pi transporter genes *OsPT2* and *OsPT8* in shoots [53]. In contrast, under Zn deficiency, a bZIP23 transcription factor-dependent *LPCAT1* gene, which encodes the lyso-phosphatidylcholine (PC) acyltransferase 1 (LPCAT1) that converts lyso-phosphatidylcholine (LPC) to phosphatidylcholine (PC), serves as the key determinant of Pi accumulation in leaves of *Arabidopsis* [58]. On the other hand, although some researchers suggest the potential involvement of Pi-starvation signaling regulator PHR1 in the integration of Pi–Zn nutrient homeostasis as it regulates the expression of *AtZIP2* and *AtZIP4* in *Arabidopsis* [51,54], whether Pi deficiency triggers Zn uptake and accumulation in roots of crop species via members of the ZIP transporter group remains to be elucidated *in planta*.

More than 70% of terrestrial vascular plants can form mutualistic associations with the obligate biotrophic soil-borne arbuscular mycorrhizal fungi (AMF) from the subphylum Glomeromycotina in the Mucoromycota [59,60,61]. The beneficial associations between host roots and AMF are referred to as arbuscular mycorrhizas (AM) and are ancient and widespread in terrestrial ecosystems [59]. During AM symbiosis, AMF provide mineral nutrients and water to the host plants, and in return obtain fatty acids and sugars to complete their obligate life cycle [62,63]. The symbiosis may not only enhance the acquisition of macronutrients such as Pi, N, K, and S to the host plant [64,65,66,67,68,69] but also facilitates the uptake of micronutrients such as Zn, Fe and Cu in the soil-AMF-plant continuum [70,71,72,73,74,75]. Besides enhancing plant mineral nutrition, AMF are capable of alleviating heavy metal toxicity such as Zn, Fe, Cu, Mn, and Cd to their host plants [20,76]. In Zn-contaminated soil, AMF can exert protective effects on plants against Zn over-accumulation [77,78]. Recently, several studies have also demonstrated that AMF play a critical role in alleviating ZnO NPs-induced toxicity to plants growing in ZnO NPs-polluted soil [79,80]. For example, AM alleviate ZnO NPs-induced phytotoxicity by decreasing Zn accumulation in maize plants and Zn partitioning to shoot [79].

Therefore, the dual benefit of AMF protects host plants from experiencing both deficient and toxic soil Zn stresses [78,81]. However, the molecular mechanisms by which AM symbiosis modulates Zn uptake and homeostasis from AM fungus to the plant and how this symbiosis coordinately regulates both Pi and Zn homeostasis in plants are difficult to be uncovered because of the close interconnection between Pi and Zn nutrients in mycorrhizal plants [78,82,83,84]. The interactions of Pi and Zn nutrients during AM symbiosis have also long been recognized at the physiological level and extensively reviewed [52,84], and this crosstalk between Pi and Zn nutrients in mycorrhizal plants can be concluded as follows: higher Pi application reduces Zn concentration in the roots or shoots of host plants [83,85,86], whereas Zn excess decreases Pi content of shoots during AM symbiosis [81]. Nevertheless, the molecular basis of the double interactions between Pi and Zn homeostasis in AM symbiosis remains largely unknown.

In this study, we broaden the current understanding of the Pi and Zn crosstalk in Chinese milk vetch (*Astragalus sinicus*) and pay more attention to a novel *A. sinicus ZIP2* gene that participates in the interconnection between Pi and Zn homeostasis in plants. We thus focus on the functional characterization of this *AsZIP2* from *A. sinicus* and show that it modulates root Zn over-accumulation under Pi starvation or Zn excess conditions. This study also presents insights into the contribution of AM to plant tolerance to heavy metal Zn through the suppression of *AsZIP2* expression.

## 2. Materials and Methods

### 2.1. Plant and AM Fungus Materials and Growth Conditions

*Astragalus sinicus* cultivar Xinyang 1 was used in this study. Seeds of *A. sinicus* were surface sterilized in 75% ethanol for 10 min, then washed with sterile water, and followed by rinsing in 3% sodium hypochlorite for 10 min [87]. After five washes, the sterilized seeds of *A. sinicus* were immersed in sterile water for 24 h, the treated seeds were then sown on 0.6% plant agar plates for germination in the dark. Seedlings were grown in a sterilized growth chamber held at 24 °C with a 16-h day and 18 °C with an 8-h night cycle. After 7 days, the seedlings were transferred into the pots containing the sterile sands with a modified Long Ashton (mLA) nutrient solution [88].

AM fungal symbiont *Rhizophagus irregularis* DAOM 197198 (formerly known as *Glomus intraradices* Schenck & Smith DAOM 197198) was used as the inoculator. The germinating spores (about 400 spores/seedling) were used for the inoculation of *R. irregularis* in roots of 14-day-old *A. sinicus* seedlings. The mycorrhizal plants were harvested at 3-, 4-, 5-, or 6-weeks post-inoculation. Parts of the root system from each mycorrhizal plant were selected under a stereomicroscope on the basis of the presence of external mycelium. These root segments were mixed together and then divided into two samples, one part used for the estimation of mycorrhizal levels, and the other for total RNA extraction.

### 2.2. Phosphate and Zinc Treatments

The *A. sinicus* plants were grown in the sand culture system treated with different nutrient elements as follows; (1) for single Pi treatment, 14-day-old seedlings of *A. sinicus* inoculated with or without *R. irregularis* were applied with the indicated Pi concentrations (30, 65, 200, 300, 1000 μM) for 42 days; (2) For individual Zn treatment, 14-day-old seedlings of *A. sinicus* inoculated with or without *R. irregularis* were cultured in sand media treated with 0.5, 1, 10, or 50 μM Zn for 42 days; (3) For Pi–Zn interaction in plants, *A. sinicus* colonized by *R. irregularis* and treated with 300 µM Pi and 50 μM Zn (+Pi+Zn), 300 µM Pi and 0.5 μM Zn (+Pi-Zn), 30 µM Pi and 50 μM Zn (-Pi+Zn) or 30 µM Pi and 0.5 μM Zn (-Pi-Zn). The standard concentrations of Pi and Zn nutrients in the complete mLA solution were defined as 300 µM NaH_2_PO_4_ and 1 µM ZnCl_2_, respectively.

All the plants were watered weekly with the mLA solution with the indicated Pi and Zn concentrations as described above. Nonmycorrhizal (NM) and arbuscular mycorrhizal (AM) plants were collected at 42 days after treatment. Roots and shoots were harvested separately and immediately frozen in liquid nitrogen, then stored at −80 °C for subsequent analysis.

### 2.3. AsZIP2 Gene Cloning and Sequencing

Based on the conserved amino acid sequences of *A. thaliana* and *M. truncatula* ZIP2 transporters [45], we designed the degenerate oligonucleotides (see Supplementary Table S1): forward (*AsZIPF1*), 5′-CAAAACCCTAAAGTCAAC-3′, which encodes KTLKST; and reverse (*AsZIPR1*), 5′-TCAATCCCAAATCATGACAAC-3′, which is antisense for VVMIWD. Subsequently, normal PCR was performed using genomic DNA from leaves of *A. sinicus*, resulting in a 1005 bp amplified DNA segment encoding a putative ZIP family Zn transporter, defined as AsZIP2. The 5′ and 3′ regions of this putative Zn transporter gene were cloned through the classic RACE methods [89,90], high-efficiency thermal asymmetric interlaced PCR (TAIL-PCR) [91], and inverse PCR (iPCR) [92]. The 5′ RACE and 3′ RACE experiments were conducted using the classic RACE protocols [89,90]. For TAIL-PCR, four longer AD (LAD) primers of 33 or 34 nucleotides were designed as reported previously [91]. For iPCR, 3 µg of total DNA isolated from *A. sinicus* leaves was digested with *Bgl*II or *Spe*I, then self-ligated and used for subsequent nested-PCR experiments. All the PCR products were cloned into the pMD^®^18-T vector (TaKaRa, Dalian, China) and sequenced. The corresponding primer sequences are listed in Supplementary Table S1.

### 2.4. Gene Expression Analysis

Total RNA was isolated from *A. sinicus* using the HP Plant RNA Kit (OMEGA, Cat. No. R6837-01, 50 preps) following the manufacturers’ instructions. The first strand of cDNA was synthesized from 1 µg of total RNA with the HiScript^®^ III RT SuperMix for qPCR (+gDNA wiper) kit (Vazyme, Nanjing, China), according to the manufacturer’s instructions. qRT-PCR experiments were performed in a 96-well Real time PCR system instrument (BioRed, Hercules, CA, USA). The reactions for gene expression were performed with three biological replicates. The *AsActin* gene from *A. sinicus* was used as an internal control. The fold change was calculated from equation 2^−ΔΔCt^ [93]. A list of gene-specific primers used for qRT-PCR is given in Supplementary Table S1.

### 2.5. Plasmid Constructs

Gateway vectors [94,95] were used in this study for *AsZIP2* promoter activity, *AsZIP2-RNAi*, and subcellular localization analyses. A 1525 bp promoter fragment of *AsZIP2* was cloned from the genomic DNA of *A. sinicus* using the specific primers *AsZIPPF* and *AsZIPPR* (see Supplementary Table S1). The PCR-amplified product was subsequently introduced into the pDONR221 (Invitrogen) and then recombined into the upstream of GUS reporter gene in the binary vector pKGWFS7.0 for tissue localization in *A. sinicus* roots. In pKGWFS7.0, the DsRed marker is under the control of the *Arabidopsis Ubiquitin10* promoter. To generate *AsZIP2-RNAi* lines, a 226-bp fragment of *AsZIP2* 5′UTR with a 35-bp coding sequence was PCR-amplified with the *AsZIP2-Ri-5′-F* and *AsZIP2-Ri-5′-R* primers (see Supplementary Table S1), subcloned into the pDONR221 vector for sequencing, and subsequently recombined into the binary vector pK7GWIWG2D(II)-RootRed [94]. The corresponding empty vector without target RNAi region, named Cheap, was used as the control.

The overexpression (OE) construct for *AsZIP2* was created using the binary vector pBI121-GFP as described previously [79]. For subcellular localization analysis in the plant cells, the Open Reading Frame (ORF) of *AsZIP2* was PCR-amplified and recombined into the Gateway vector pK7FWG2.0 with the eGFP reporter under the control of the 35S promoter to generate the ZIP2::eGFP fusion. Transient co-expression of the AsZIP2-eGFP construct with the plasma membrane marker CERK1-DsRed [96] in the abaxial epidermal cells of *Nicotiana benthamiana* was performed by the agroinfiltration protocol described previously by Pan et al. (2016) [97].

### 2.6. Plant Transformation

The *Agrobacterium rhizogenes* K599-mediated *A. sinicus* transformation was essentially performed as described previously [98]. Transgenic *A. sinicus* hairy roots were detected by the observation of red fluorescence from the DsRed reporter using a fluorescence stereomicroscope (Nikon SMZ18) [87]. Positive transformed lines were selected for further studies.

### 2.7. Microscopy

Confocal microscopic analysis of the protein subcellular localization was conducted with a laser scanning confocal microscope with a 63 × water-immersion objective (Zeiss 780). The excitation wavelengths were 488 and 543 nm for GFP and mCherry, respectively, while the emitted wavelengths were 500–550 nm for GFP, and 565–615 nm for mCherry. The fluorescence stereomicroscope (Nikon SMZ18) was used to select the hairy root with DsRed fluorescence. To observe the tissue localization of *AsZIP2* promoter activity, live NM and AM roots were mounted on slides and imaged using a Nikon ECLIPSE Ni fluorescence microscope, which was also used for recording the arbuscular mycorrhizas in roots after the WGA-Alexa Fluor 488 (WGA488) staining.

### 2.8. GUS Expression Analysis

Histochemical GUS assay of *A. sinicus* hairy roots was carried out as described previously [87,95]. The *A. sinicus* hairy roots colonized with or without *R. irregularis* were treated with different Pi and Zn concentrations as shown in Figures 3e–j and 4g–i. The collected roots with red fluorescence were incubated in the GUS buffer [87] at 37 °C in the dark. After staining and washing, roots were observed under an optical microscope (Olympus BX51).

### 2.9. Yeast Transformation, Growth and Zn Uptake Assay

Heterologous expression of *AsZIP2* in yeast mutant strain was conducted as described previously [99,100], with minor modifications. In Brief, *Saccharomyces cerevisiae zrt1zrt2* mutant ZHY3 (*MATα ade6 can1 leu2 his3 trp1 ura3 zrt1::LEU2 zrt2::HIS3*) [99,100], which is defective in two ZIP family transporters for Zn uptake, and wild-type strain BY4741 (*MATα his3 leu2 met15 ura3*) were used for complementation analysis. The shuttle vector pFL61 (kindly provided by Dr. Luisa Lanfranco, University of Turin, Italy) was used for yeast expression vector [101]. The commercial Zn-free YNB medium (Sigma-Aldrich, St. Louis, MO, USA) without uracil was purchased for growth analysis. To confirm the role of *AsZIP2* in yeast cells, the full-length coding sequence of this gene was introduced into the *Not*I site of pFL61 driven by the *PGK* promoter to generate the *pFL61-AsZIP2* construct. On the other hand, the full-length sequence of *AtZIP2* was isolated from *A. thaliana* and cloned into the pFL61, resulting in the *pFL61-AtZIP2* construct as a positive control, whereas the pFL61 empty vector served as a negative control. These resulting plasmids were further validated by sequencing.

The *S. cerevisiae* strains ZHY3 and BY4741 were transformed and cultivated for complementation test as previously described [23,102]. The transformed strains ZHY3 and BY4741 were grown on SD medium without uracil (pH 5.8), containing 10 mM 2-(N-morpholino) ethanesulfonic acid (MES) and supplemented with 5, 50, or 250 μM ZnCl_2_. This Zn-restricting SD medium contained 1 mM EDTA. In the growth test, 5-μL drops of culture dilutions from 0.1 to 0.0001 were spotted onto the selective medium and grown for 2 days at 30 °C.

To further confirm facilitation of zinc uptake by AsZIP2 in yeast cells, we performed the zinc uptake assay with *S. cerevisiae* mutant *zrt1zrt2* expressing *AsZIP2*, or *AtZIP2* in pFL61. To measure the yeast Zn concentration, cells grown to OD_600 nm_ = 0.6 in liquid SD medium (containing 2% glucose, 10 mM MES, 1 mM EDTA, pH 5.8) supplemented with 5, 50, or 250 μM ZnCl_2_ were harvested and analyzed using inductively coupled plasma-optical emission spectroscopy (ICP-OES, 710-ES, VARIAN, Palo Alto, CA, USA).

### 2.10. Mycorrhizal Colonization Analysis

The digested AM roots of *A. sinicus* were stained with 5.0 μg/mL WGA488 (Invitrogen, Carlsbad, CA, USA) for 30 min at 37 °C, washed three times for 5 min in 1 × HBSS [87], and the levels of mycorrhizal colonization were quantified according to Trouvelot et al. (1986) [103] using MYCOCALC (http://www2.dijon.inra.fr/mychintec/Mycocalc-prg/download.html, accessed on 1 November 2020).

### 2.11. Elemental Analysis

To measure total P and Zn concentrations in *A. sinicus*, the collected roots and shoots were placed in a vacuum lyophilizer (Christ, Germany) until completely dry. Weighed samples (about 30 mg) were powdered by a grinder and then digested in 1 mL of 6 M nitric acids at 90 °C for 1 h [87]. The digest was diluted by the addition of 1 mL of ddH_2_O. After filtration, a further dilution step (1:6) was essentially performed. The total P and Zn concentrations of the digests were determined using the ICP-OES (710-ES, VARIAN, USA). Pi and Zn standard solutions for calibration were from the Test Center at South China Agricultural University in Guangzhou of China.

### 2.12. In Silico Analysis

Analysis of the full-length amino acid sequences of AsZIP2 and other plant ZIP2 transporters was conducted utilizing the following programs: NCBI Blast Genomes Server for the homology searches for plant ZIP2 proteins (www.ncbi.nlm.nih.gov, accessed on 1 July 2021), Clustal Omega for multiple sequence alignment (www.ebi.ac.uk/Tools/msa/clustalo/, accessed on 1 July 2021), The TOPCONS program (http://topcons.cbr.su.se/, accessed on 1 July 2021) was used to predict transmembrane domains, membrane orientation and signal peptide. The PROSITE web server was used for searching protein sequence motifs (www.genome.jp/tools/motif/, accessed on 1 July 2021). The PlantCARE database (http://sphinx.rug.ac.be:8080/PlantCARE/, accessed on 1 August 2021) was used to find the *cis*-acting regulatory DNA elements of *AsZIP2* promoter sequence [104]. The P1BS-motif was screened in the promoter regions of the plant ZIP2 subfamily genes by DNA-pattern matching analysis (http://rsat.ulb. ac.be/rsat/, accessed on 1 August 2021).

### 2.13. Phylogenetic Analysis

Phylogenetic analysis was analyzed in MEGA7.0 [105]. The well-aligned amino acid sequences by Clustal Omega were loaded into the MEGA7.0 program for phylogenetic analysis. The evolutionary history was inferred using the Neighbor–Joining method. The bootstrap consensus tree inferred from 1000 replicates is taken to represent the evolutionary history of 63 plant ZIP family proteins tested. The evolutionary distances were computed using the Poisson correction method. The bootstrap values are shown at the nodes. The accession numbers of the plant ZIP family transporters used in this study are provided in Supplementary Table S2.

### 2.14. Statistical Analysis

Statistical significance of differences between averages was estimated by analysis of variance (ANOVA) using SPSS16 software (SPSS Inc., Chicago, IL, USA). The Duncan’s multiple range test was used for comparing more than two datasets, whereas the Student’s *t*-test was used for pairwise comparisons between the control and *AsZIP2-OE* or *AsZIP2-RNAi* lines. The data were presented as the mean ± SD of different replicates, except for the OE and RNAi experiments. Means with different letters indicate the statistical differences at *p* < 0.05, while the asterisks above the error bars represent means that were statistically different at: *, *p* < 0.05; **, *p* < 0.01; ***, *p* < 0.001. All microscopy images shown are representative of AM fungal structures observed in multiple independent plant lines.

### 2.15. Accession Numbers

Sequence data from this article can be found in the plant genome and GenBank libraries under the following accession numbers for *A. sinicus* and model plant ZIP2 subfamily proteins: *A. sinicus* AsZIP2 (QYE52148), *M. truncatula* MtZIP2 (XP_003597387.1), *Lotus japonicus* LjZIP2 (AFK49261.1), *Glycine max* GmZIP2 (XP_003543520.3), *Solanum lycopersicum* SlZIP2 (NP_001234349.1), *Populus trichocarpa* PtZIP2 (XP_002312231.2), *A. thaliana* AtZIP2 (NP_200760.1), *O. sativa* OsZIP2 (XP_015628224.1).

## 3. Results

### 3.1. Zn Concentration Is Over-Accumulated in Pi-Starved Astragalus sinicus, yet Reduced by Arbuscular Mycorrhizal Colonization

**Figure 1:**
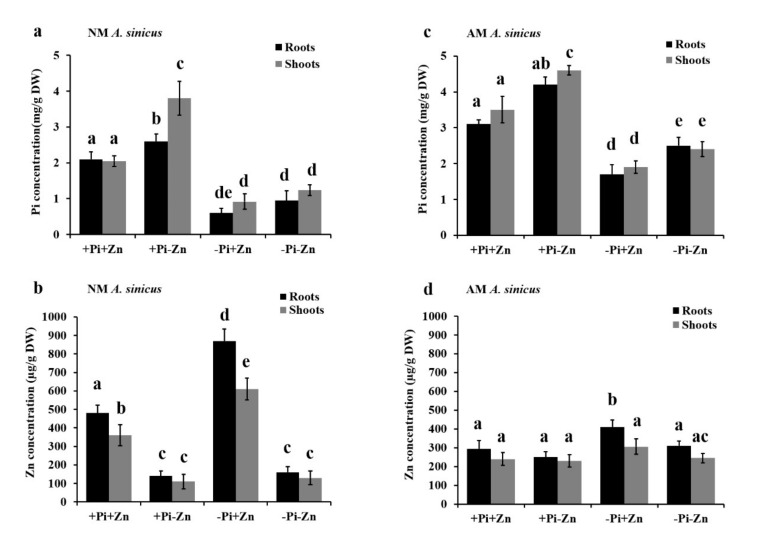
Physiological analysis of phosphorus and zinc interaction in *A. sinicus* plants. *A. sinicus* plants were grown in pot cultures treated with 300 µM Pi and 50 μM Zn (+Pi+Zn), 300 µM Pi and 0.5 μM Zn (+Pi-Zn), 30 µM Pi and 50 μM Zn (-Pi+Zn) or 30 µM Pi and 0.5 μM Zn (-Pi-Zn). Pi and Zn concentrations were quantified in roots (black) and shoots (gray) of 56-d-old NM (**a**,**b**) or AM (**c**,**d**) plants. *A. sinicus* roots colonized by *R. irregularis* at 42 dpi. (**a**,**c**) Total P concentration in *A. sinicus*; (**b**,**d**) Zn concentration in *A. sinicus*. Error bars indicate the standard deviation from three biological replicates. The different letters are statistically significant differences among treatments at *p* < 0.05, based on Duncan’s multiple range test. DW, Dry weight.

To gain insights into the physiological base of interaction between Pi and Zn nutrients in *A. sinicus*, 14-day-old-seedlings inoculated with or without *R. irregularis* were grown in cultures supplemented with either high Pi and high Zn (+Pi+Zn, 300 µM Pi and 50 μM Zn), high Pi and low Zn (+Pi-Zn, 300 µM Pi and 0.5 μM Zn), low Pi and high Zn (-Pi+Zn, 30 µM Pi and 50 μM Zn), or both low Pi and Zn (-Pi-Zn, 30 µM Pi and 0.5 μM Zn). Subsequently, we determined the total P and Zn concentrations of root and shoot tissues from 56-d-old NM and AM plants under four different nutrient conditions as already mentioned by the ICP-OES analysis (Figure 1). As expected, low Zn led to a remarkable increase in total P concentration in *A. sinicus* plants (Figure 1a), conversely, Pi starvation resulted in Zn over-accumulation in roots and shoots of *A. sinicus* (Figure 1b). This result confirms the antagonistic interaction between Pi and Zn nutrients in *A. sinicus* plants. On the other hand, during AM symbiosis, under nutrient deficiencies (low Pi and/or low Zn), both total P and Zn concentrations were significantly enhanced in AM *A. sinicus* plants compared with those in NM plants (Figure 1a,c, *p* < 0.05), suggesting a positive effect of AM fungus *R. irregularis* on plant Pi and Zn uptake and homeostasis during nutrient deficiencies. During AM symbiosis, Zn deficiency also induced P accumulation in *A. sinicus* plants (Figure 1c). However, under high Zn treatment, low Pi did significantly promote Zn uptake in roots during AM symbiosis (Figure 1d), whereas Zn accumulation in *A. sinicus* was significantly decreased by arbuscular mycorrhizal colonization under such conditions in relation to NM plants (Figure 1b,d, *p* < 0.05). Additionally, we determined the effects of Pi and Zn treatments on fungal colonization in roots of *A. sinicus*. The results indicate that these four nutrient treatments did not significantly affect the total colonization and arbuscular mycorrhiza in roots (Figure S1). Overall, these results reveal that Zn concentration is over-accumulated in Pi-starved *A. sinicus*, but significantly reduced by arbuscular mycorrhizal colonization. These findings raise the open question of whether non-negligible Zn transporters were largely regulated by Pi starvation or arbuscular mycorrhization, allowing Zn over-accumulation in NM plants or Zn alleviation in AM plants.

### 3.2. Identification of the AsZIP2 Gene in A. sinicus

The conserved amino acid sequences of *A. thaliana* and legume plant ZIP transporters [1,45] allowed us to isolate the specific coding sequence that corresponds to putative ZIP transporter in *A. sinicus*. Using the RACE and inverse PCR methods, the full-length sequence of *AsZIP2* was obtained (The GenBank accession number is MZ636517). In silico analysis showed that *AsZIP2* consists of two exon and one intron (Figure S2a), and comprises 339 amino acids (QYE52148). The ORF of *AsZIP2* is predicted to encode a transmembrane protein containing 8 TM domains separated by a large variable hydrophilic region between TMD III and TMD IV, and a signal peptide at the amino terminus, based on the TOPCONS web server (http://topcons.cbr.su.se/, accessed on 1 July 2021) (Figure S2b). Further sequence analysis showed that the consensus sequence within TMD IV of AsZIP2 fits the pattern typical for the ZIP family of proteins (VALCFHSVFEGIAIG) [106,107]. This structure is also consistent with the topology of Zn transporters of the ZIP family in plants [1,45].

To further search the putative *cis*-regulatory elements in the promoter region, we analyzed a 1613 bp upstream sequence starting from the start codon ATG of *AsZIP2* using the PlantCARE database. Through this promoter analysis, we found a *cis*-element P1BS (GTATATGC) that was essentially targeted by the PHR1 transcription factor [108] (Figure S3), indicating that the transcription of *AsZIP2* may be directly dependent on the PHR1 factor in *A. sinicus*. Furthermore, the ZIP2 subfamily genes of other plant species also contain at least one P1BS motif in their promoter regions (Figure S3). This finding indicates that *A. sinicus AsZIP2* (including other *ZIP2* genes) may be involved in Pi starvation signaling in plants.

### 3.3. ZIP2 Zinc Transporters Are Conserved across Dicotyledons and Monocotyledons

**Figure 2:**
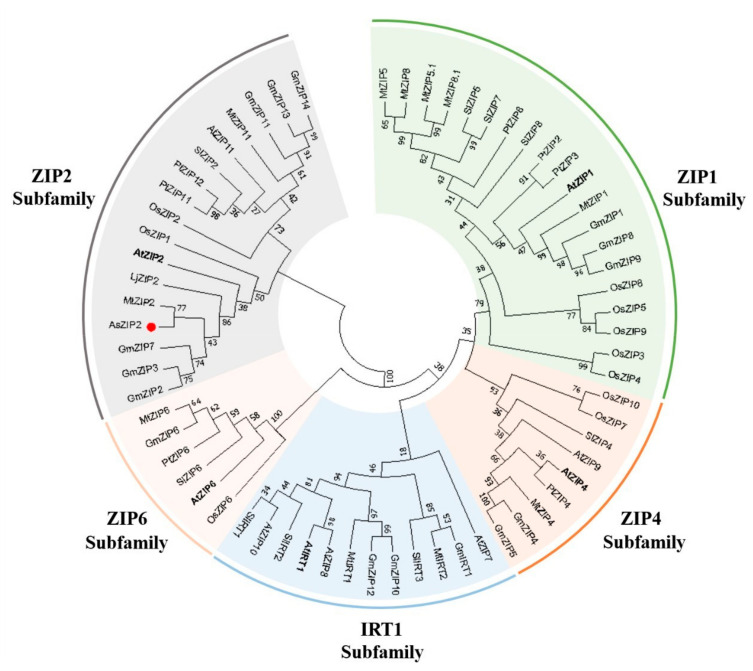
Evolutionary relationship among AsZIP2 in *A. sinicus* and ZIP family zinc/iron transporters in different plant species. The evolutionary history was inferred using MEGA7.0 program with the Neighbour–Joining method (Kumar et al., 2016). The evolutionary distances were computed using the Poisson correction method. All ambiguous positions of the 63 amino acid sequences were removed for each sequence pair. Plant ZIP family proteins tested were classified as five subfamilies based on sequence similarity. The branch marked with a red circle represents the Zn transporter AsZIP2 of *A. sinicus* in this study. The accession numbers and plant species names are provided in Supplementary Table S2.

To determine the evolutionary relationships between AsZIP2 and other members of the ZIP family in different plants, we constructed an unrooted phylogenetic tree for 63 proteins of the ZIP family from diverse plants using the MEGA7.0 software [105]. As shown in Figure 2, these plant ZIP family transporters can be divided into five groups based on the conservation among ZIP proteins, consisting of one ZIP1 subfamily, one ZIP2 subfamily, one ZIP4 subfamily, one ZIP6 subfamily and one IRT1 subfamily.

Among them, the ZIP2 subfamily consists of the transporters from the dicots and monocots (such as *O. sativa*) and can be further classified into two clusters (Figure 2). In the ZIP2 subfamily, the AsZIP2 Zn transporter from *A. sinicus* is closely related to ZIP2 in legumes and Arabidopsis as well as OsZIP1 in *O. sativa*, but forms a distinct subgroup from the ZIP1 subfamily, ZIP4 subfamily, ZIP6 subfamily, and IRT1 subfamily. Moreover, the early reported MtZIP2 transporter from *M. truncatula* clusters closely together with AsZIP2 within the ZIP2 subfamily [40], indicating that these two transporters may have similar functions. In conclusion, the ZIP2 Zn transporters are conserved across dicotyledons and monocotyledons.

### 3.4. The A. sinicus AsZIP2 Gene Is Highly Expressed in Response to Pi Starvation

**Figure 3:**
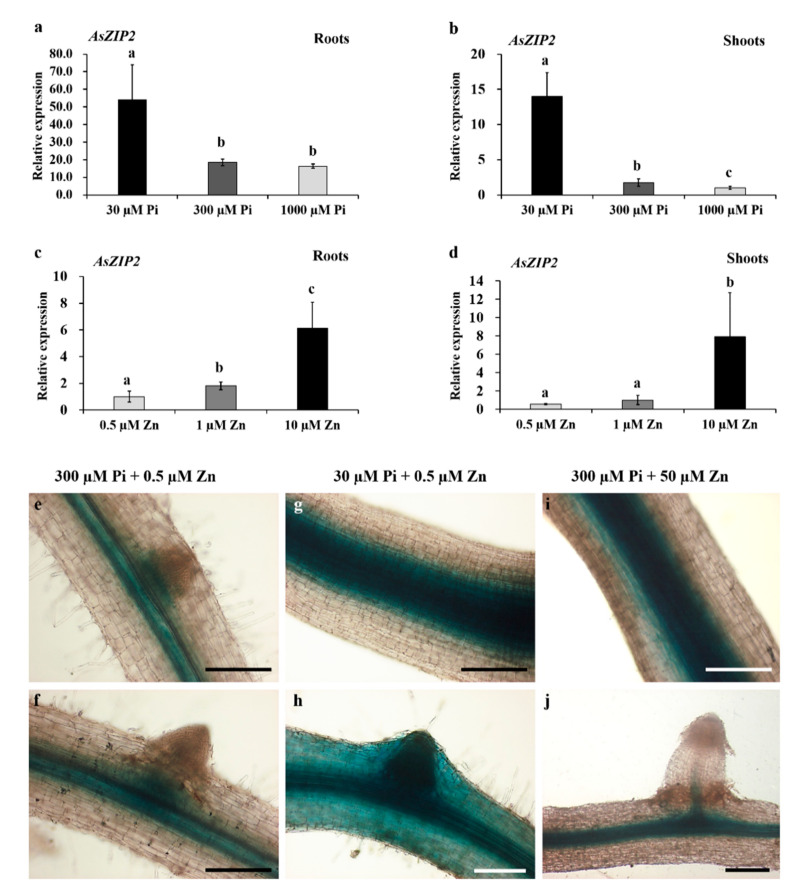
The expression patterns of *AsZIP2* in *A. sinicus*. (**a**–**d**) Relative expression of *AsZIP2* in response to external Pi and Zn treatments, as determined by real time qRT-PCR. 56-day-old *A. sinicus* plants were grown in sand cultures supplied with the indicated nutrient concentrations. *AsActin* from *A. sinicus* served as an endogenous control for normalization. Data are shown as means ± SD of three biological replicates. Averages with the different letters are significantly different at *p* < 0.05, based on Duncan’s multiple range test. (**e**–**j**) Histochemical localization in *pAsZIP2::GUS* transgenic *A. sinicus* hairy roots treated with different Pi and Zn concentrations. Positive GUS staining of the primary (**e**,**g**,**i**) and lateral (**f**,**h**,**j**) roots treated with 30 or 300 µM phosphate, grown under low Zn (0.5 µM) or moderately high Zn (50 µM) conditions described in each panel. (**e**) The primary root with a primordium; (**g**,**i**) The strong GUS staining of the central cylinder of primary roots. (**h**) The GUS signal is also present in the root epidermis, cortex, and primordium during Pi starvation. Scale bars, 100 µm.

To examine the expression profiles of the AsZIP2 Zn transporter in response to external different Pi concentrations, the relative expression of *AsZIP2* at the transcriptional level was characterized in root and shoot tissues of *A. sinicus* under various Pi levels. The quantitative RT-PCR showed that *AsZIP2* was highly expressed in the roots and shoots of *A. sinicus* grown under Pi deficiency (Figure 3a,b).

To extend the qRT-PCR results, we further determined the tissue-specific expression pattern of *AsZIP2* in response to Pi availability in *A. sinicus* roots, and generated hairy roots expressing a *pAsZIP2::GUS* fusion construct. The *A. sinicus* with hairy roots carrying the *pAsPT5::GUS* fusion were grown in sand cultures supplemented with 30 or 300 µM Pi. The results of GUS staining revealed that *AsZIP2* was predominantly expressed in the central cylinder of primary roots and the base of lateral root primordium under control Pi (300 µM) conditions (Figure 3e,f). Noticeably, the GUS activity driven by the promoter of *AsZIP2* was strongly expressed in the central cylinder of primary roots (Figure 3g), while the promoter activity of *AsZIP2* was also present in the epidermal, cortical, and stele cells of lateral roots (Figure 3h) during Pi starvation (30 µM). Taken together, these results indicate that *AsZIP2* may be involved in Zn uptake in *A. sinicus* roots during Pi starvation.

### 3.5. High Zn Supply Induces AsZIP2 Expression

A previous report showed that a ZIP2 Zn transporter from *M. truncatula* was up-regulated in roots by Zn fertilization [45]. Interestingly, AsZIP2 is closely related to the MtZIP2 transporter from the evolutionary relationship (see Figure 2), which may suggest their similar expression pattern in Zn response. First, we examined the expression of *AsZIP2* in response to external Zn concentrations by qRT-PCR analysis using total RNA from roots and shoots of *A. sinicus* plants grown on sand cultures containing 300 µM Pi, and supplied with the Zn concentrations at the range of 0.5–10 µM for 42 days. As shown in Figure 3c,d, the transcription of *AsZIP2* was responsive to the exogenous high Zn supply in the tissues examined.

As mentioned above, GUS staining in *proAsZIP2::GUS A. sinicus* hairy roots showed that *AsZIP2* is mainly expressed in the stele cells in roots under control conditions (see Figure 3e). To further examine the activity of *AsZIP2* promoter in *A. sinicus* roots exposed to high Zn concentration (50 μΜ), the *proAsZIP2::GUS* fusion construct was transformed into *A. sinicus* hairy roots, which were grown in cultures containing 300 μM Pi and 50 μM Zn. The result reveals that, under high Zn condition, the *proAsZIP2* activity was obviously increased in the stele cells in roots of *A. sinicus* (Figure 3i,j) when compared with that in low Zn-treated roots (Figure 3e,f).

Taken together, these results indicate that *AsZIP2* is also induced in *A. sinicus* roots by high Zn supply.

### 3.6. Expression Analyses of AsZIP2 Involved in Pi and Zn Interaction in A. sinicus

**Figure 4:**
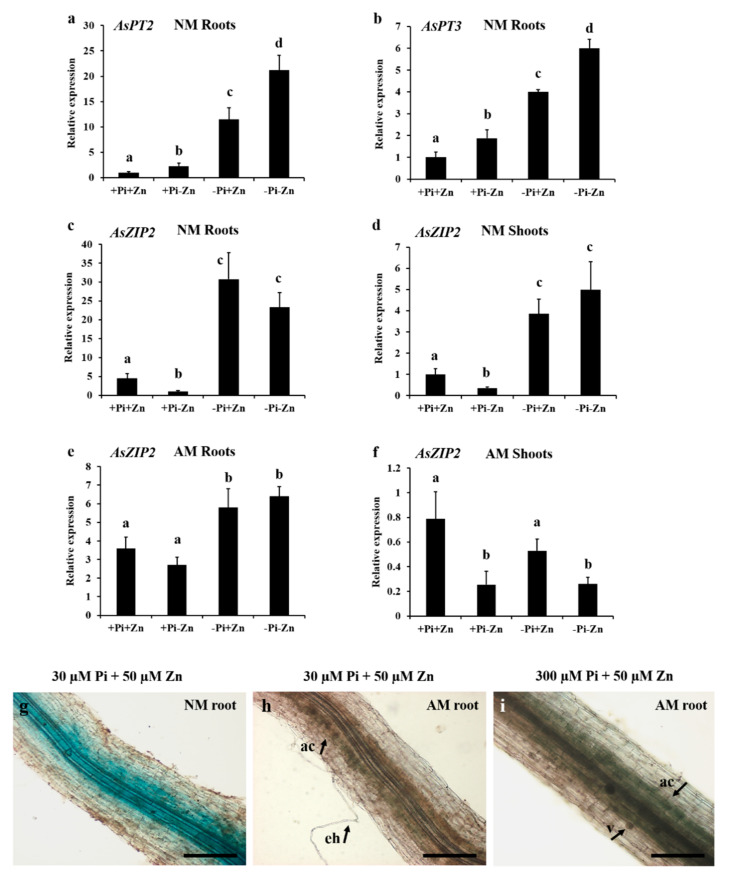
Expression profiles of the two PHT1 family members *AsPT2/3* and one ZIP family member *AsZIP2* in *A. sinicus* in response to Pi and/or Zn availabilities. *A. sinicus* plants were grown in cultures treated with 300 µM Pi and 50 μM Zn (+Pi+Zn), 300 µM Pi and 0.5 μM Zn (+Pi-Zn), 30 µM Pi and 50 μM Zn (-Pi+Zn) or 30 µM Pi and 0.5 μM Zn (-Pi-Zn). (**a**–**f**) Fourteen-day-old *A. sinicus* seedlings were colonized by *R. irregularis* at 42 dpi, and roots and shoots of 56-d-old plants were collected separately with transcription levels of target genes quantified by real-time qRT-PCR. (**a**–**d**) Gene expression in NM *A. sinicus* plant tissues; (**e**,**f**) Gene expression in *A. sinicus* plants under AM conditions. The *AsActin* gene for *A. sinicus* was used as the house-keeping gene for normalization. Error bars mean standard deviation from three biological replicates. The different letters are statistically significant differences among treatments at *p* < 0.05, based on Duncan’s multiple range test. (**g**–**i**) GUS staining of *pAsZIP2::GUS* transgenic NM (**g**) and AM (**h**,**i**) roots of *A. sinicus* grown under low Pi and/or moderately high Zn conditions as indicated. *A. sinicus* roots colonized by R. irregularis at 42 dpi. NM, nonmycorrhizal; AM, arbuscular mycorrhizal; ac, arbuscule-containing cells; eh, extraradical hyphae; v, vesicles. Scale bars, 100 µm.

Since earlier studies report that some Pi transporters of the plant PHT1 family were highly induced in plants during Zn deficiency [48,56], here we also determined whether the *A. sinicus* Pi transporter genes were regulated in response to Zn deficiency response. First, we evaluated the expression profiles of three PHT1 Pi transporter genes in *A. sinicus*, referred as to *AsPT2*, *AsPT3*, and *AsPT4* [109]. As expected, *AsPT2* and *AsPT3* were significantly up-regulated in NM roots of *A. sinicus* during Pi starvation (Figure 4a,b), while the expression of *AsPT4*, which also served as an AM symbiotic marker gene [109], was induced in mycorrhizal roots under low Pi conditions (Figure S4). Under Zn deficiency, there was an obvious increase in the transcription of *AsPT2* and *AsPT3* in *A. sinicus* roots, whereas *AsPT4* was not up-regulated in response to Zn deficiency in mycorrhizal roots (Figure 4a,b and Figure S4). These results show that Zn deficiency has a partial effect on the induction of PHT1 family Pi transporters, resulting in the Pi accumulation in *A. sinicus* plants.

Next, we focused on the ZIP family gene *AsZIP2*, which was highly expressed in roots (Figure S5). Interestingly, it has been shown that transcript levels of this *AsZIP2* were strongly induced in Pi-starved root and shoot tissues of *A. sinicus* under both Zn-sufficient and Zn-deficient conditions (Figure 4c,d). Similarly, during AM symbiosis, *AsZIP2* exhibited higher expression in lower Pi status of AM plants (Figure 4e; see Figure 1c). By contrast, high Zn supply increased *AsZIP2* expression in both roots and shoots (Figure 4c,d), whereas this induction was attenuated in *A. sinicus* during AM symbiosis (Figure 4e,f, *p* < 0.05), although *AsZIP2* is slightly but significantly increased in AM *A. sinicus* under low Pi or high Zn treatment.

Taken together, the molecular analyses uncovered that *AsZIP2* may act as a linker in the interaction between Pi and Zn nutrients in *A. sinicus* plants.

### 3.7. Arbuscular Mycorrhization Represses AsZIP2 Expression in a Pi-Dependent Manner

As AM fungus facilitated the plant uptake of Pi and Zn nutrients, and the down-regulation of *AsZIP2* was examined in the roots of *A. sinicus* colonized by *R. irregularis* (see Figure 1 and Figure 4e), we thus proposed that the activity of *AsZIP2* promoter was inhibited in arbuscular mycorrhizal roots of *A. sinicus* grown under low Pi or high Zn conditions. Expectedly, in the presence of AM fungi, the expression of *AsZIP2* in the stele cells of roots appeared to be much weaker when compared with that under NM condition (see Figure 4g–i), regardless of Pi deficiency or Zn excess.

**Figure 5:**
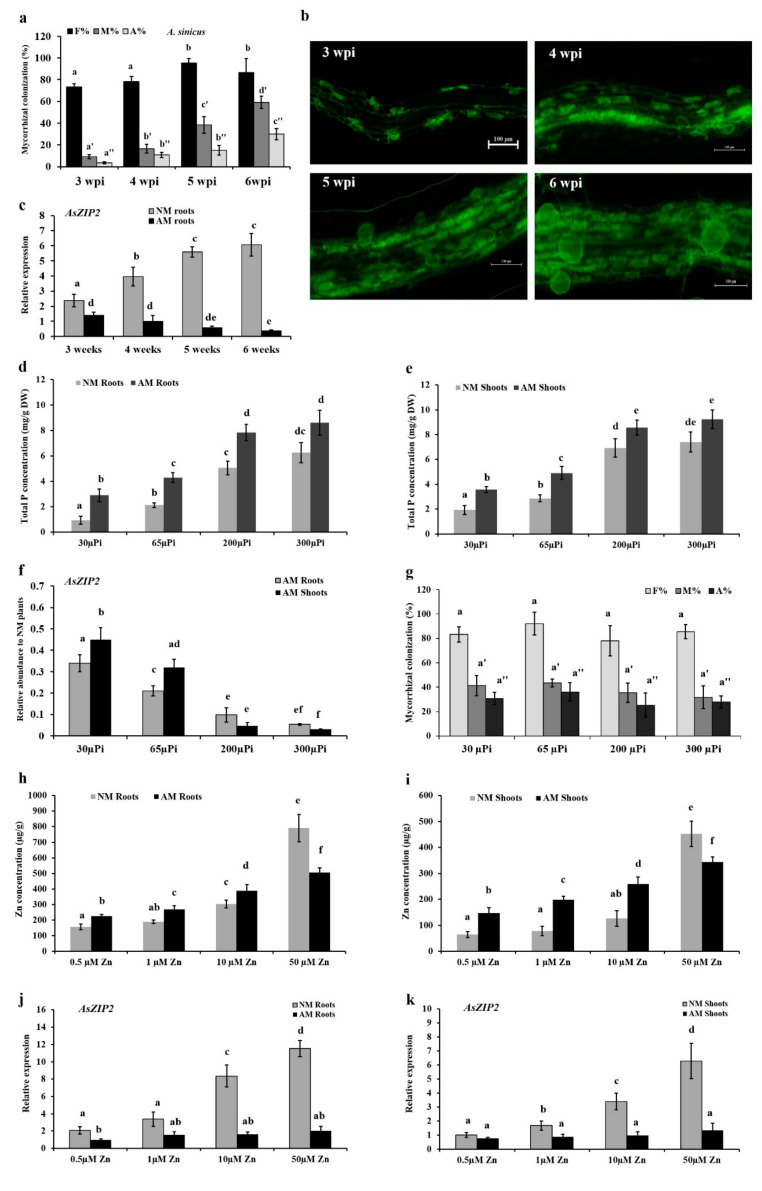
Arbuscular mycorrhizal colonization reduces the expression of Zn transporter gene *AsZIP2* in a phosphate-dependent manner. (**a**–**c**) Mycorrhization strongly represses the *AsZIP2* expression in *A. sinicus* roots inoculated with (AM) or without (NM) *R. irregularis*. (**a**,**b**) The mycorrhization in *A. sinicus* roots colonized by *R. irregularis*, grown under low Pi (30 µM) conditions at different weeks post-inoculation (wpi). (**a**) Mycorrhizal colonization levels were determined after WGA488 staining. F%, the total colonization frequency; M%, the percentage of mycorrhizal intensity; A%, the percentage of arbuscule abundance. (**b**) Fluorescence microscope images of *R. irregularis* within roots at different wpi. Scale bars, 100 µm. (**c**) The expression of *AsZIP2* in NM and AM roots of *A. sinicus* at different weeks. NM, nonmycorrhizal; AM, arbuscular mycorrhizal. (**d**–**g**) The expression pattern of *AsZIP2* in *A. sinicus* plants is dependent on the phosphate status during AM symbiosis. Total P concentrations were determined in the roots (**d**) and shoots (**e**) of NM and AM *A. sinicus* grown under the indicated Pi conditions shown. (**f**) The expression of *AsZIP2* in AM roots and shoots of *A. sinicus* in response to different Pi concentrations indicated, estimated by real-time qRT-PCR. *AsActin* from *A. sinicus* served as the internal control. (**g**) The effect of different Pi availability on arbuscular mycorrhization in *A. sinicus* roots at 42 dpi. (**h**–**k**) The transcription of *AsZIP2* is independent of the Zn status in *A. sinicus* during AM symbiosis. Zn concentrations were estimated in the roots (**h**) and shoots (**i**) of NM and AM *A. sinicus* during different Zn conditions indicated. Transcription of *AsZIP2* in the roots (**j**) and shoots (**k**) of NM and AM *A. sinicus* exposed to different Zn concentrations indicated, measured by real-time qRT-PCR. Error bars represent the SD for means of three biological replicates. Different letters indicate statistically significant differences at *p* < 0.05, based on the Duncan’s multiple range test.

We further investigate whether the colonization frequency, Pi status and Zn levels affect the expression pattern of *AsZIP2* in *A. sinicus* during AM symbiosis. First, we compared the transcript levels of *AsZIP2* with the AM fungal colonization frequency (Figure 5a) and arbuscule abundance (Figure 5b), which were gradually increased with the prolongation of colonization time. The results show a negative correlation in the expression levels of *AsZIP2* and colonization frequency (or arbuscule abundance) during AM symbiosis (Figure 5c). However, in control (NM) roots, the *AsZIP2* expression is enhanced with the growth duration increases under Pi starvation. These findings indicate that the expression levels of *AsZIP2* mirror the colonization frequency as well as arbuscule development, and are gradually repressed in roots during the mycorrhization process.

To next investigate the effect of Pi status on the expression pattern of *AsZIP2* in *A. sinicus* during AM symbiosis, we measured the total P concentration of plants grown at different external Pi concentrations as indicated in Figure 5d,e. Low Pi (30 and 65 μΜ) and moderately high Pi (200 and 300 μΜ) treated mycorrhizal plants had higher Pi levels in roots and shoots than the corresponding NM plants (Figure 5d,e). As expected, the result reveals that transcript levels of *AsZIP2* are dramatically decreased with the increased P concentration in *A. sinicus* tissues during AM symbiosis (Figure 5f). By contrast, the result of fungal colonization shows that *A. sinicus* plants grown at the range of 30–300 µM Pi concentrations exhibited similar levels of fungal frequency and arbuscule abundance (Figure 5g). This is consistent with the previously reported result by Fan et al. (2020) [87]. Therefore, these experiments indicate that the expression levels of *AsZIP2* are regulated in a Pi-starvation dependent manner during AM symbiosis.

Finally, we investigated the impact of different Zn levels on the *AsZIP2* expression in AM *A. sinicus*. As expected, low Zn (0.5 and 1 μΜ) treated AM plants showed significantly higher Zn concentrations in both root and shoot tissues than the corresponding NM plants (Figure 5h,i). By contrast, long-term exposure to high Zn (10 and 50 μΜ) conditions led to Zn over-accumulation in NM plants, whereas this inducible effect was repressed in the roots and shoots of AM plants, which had less Zn concentrations than the NM plants (Figure 5h,i). Despite these low and high levels of Zn supplies, the AM fungal colonization and arbuscule development remained unaffected within *A. sinicus* roots (Figure S6). Importantly, *AsZIP2* expression was significantly repressed in all these Zn-treated AM plants when compared with those NM plants (Figure 5j,k). These results indicate that transcription of *AsZIP2* is independent of Zn levels in *A. sinicus* during AM symbiosis, suggesting that Zn indirectly regulates *AsZIP2* expression.

Taken as a whole, the data stemming from these physiological and molecular experiments indicate that the *A. sinicus AsZIP2* expression is attenuated by arbuscular mycorrhization in a Pi-dependent manner.

### 3.8. The A. sinicus AsZIP2 Transporter Behaves as a Zinc Transporter in Yeast

To further determine whether the *A. sinicus AsZIP2* encodes a functional Zn transporter, the full-length of *AsZIP2* was inserted into the pFL61 yeast expression vector [101]. The resulting construct, *pFL61-AsZIP2*, was expressed in the yeast mutant strain ZHY3 (Δ*zrt1zrt2* mutant) [99,100], which is defective in two ZIP family Zn transporters. Subsequently, the Zn transport properties of the *A. sinicus* AsZIP2 transporter were analyzed in the heterologous yeast expression system.

**Figure 6:**
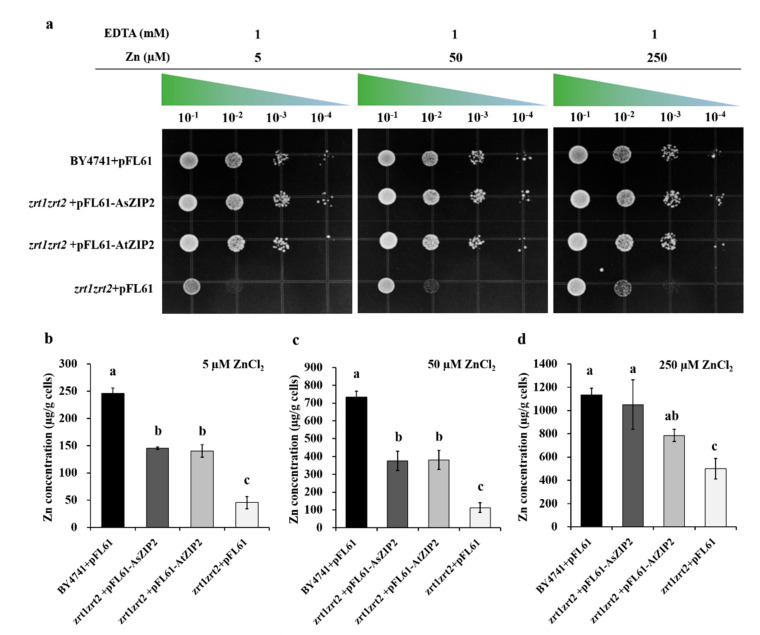
Complementation of *Saccharomyces cerevisiae* mutant *zrt1zrt2* and wild-type strain BY4741. Yeast cells expressing *AsZIP2* and *AtZIP2* in the vector pFL61 were grown under different Zn concentrations as indicated. (**a**) The wild-type strain BY4741 transformed with *pFL61* and Zn (*zrt1zrt2*) transport mutant carrying *AtZIP2* were used as the positive controls, while the mutant *zrt1zrt2* carrying *pFL61* was applied as a negative control. The mutant *zrt1zrt2* transformed with *AsZIP2* was used for validation. Transformed cells were grown on SD medium without uracil (SD/-ura) and plus 1 mM EDTA and 5, 50, or 250 µM ZnCl_2_. The gradient marks above each panel indicate cell dilutions from 10^−1^ to 10^−4^. For tests, 5-µL drops of each dilution was spotted on solid SD/-ura media and grown for 2 days at 30 °C. (**b**–**d**) Zn concentrations in the yeast mutant *zrt1zrt2* strain expressing *AsZIP2*, *AtZIP2*, or *pFL61* vector and in wild-type BY4741 with *pFL61*, grown under 5 (**b**), 50 (**c**), or 250 µM (**d**) ZnCl_2_ conditions. The Zn concentrations in yeast cells grown in SD/-ura media as shown in Figure 6b–d. The data are expressed as µg Zn per g cell dry weight. Error bars represent the SD for means of three independent experiments. Different letters indicate significant differences at *p* < 0.05, based on the Duncan’s multiple range test.

We first examined the ability of AsZIP2 transporter to complement the growth defect of the ZHY3 strain. As expected, the ZHY3 cells expressing the *pFL61-AsZIP2* or *pFL61-AtZIP2* (as a positive control) showed restored growth on lower (5 μΜ) and higher (50 and 250 μΜ) Zn media, whereas the ZHY3 strain carrying the empty vector pFL61 exhibited a weaker growth under such conditions (Figure 6a). This finding revealed that *AsZIP2* may encode a functional Zn transporter. To correlate the recovered growth with an enhanced Zn uptake of the yeast cells, we next measured the intracellular Zn concentration of the different yeast strains grown under 5, 50, and 250 μΜ Zn conditions. The data revealed that ZHY3 strain expressing *pFL61-AsZIP2* or *pFL61-AtZIP2* accumulated much more Zn concentration in cells when compared with this mutant strain carrying the pFL61 empty vector under lower Zn conditions (Figure 6b,c), whereas the Zn levels in the ZHY3 mutant strain expressing *AsZIP2* were comparable to those of the wild type strain under higher Zn conditions (Figure 6d). In conclusion, the results coming from the yeast heterologous expression indicate that the *A. sinicus* AsZIP2 transporter functions as a Zn transporter and facilitates the Zn uptake.

### 3.9. The A. sinicus AsZIP2 Gene Encodes a Plasma Membrane-Localized Transporter

**Figure 7:**
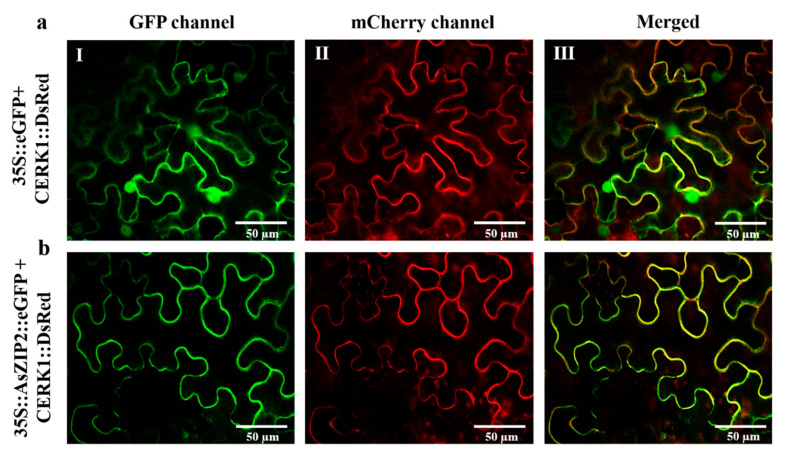
Subcellular localization of the AsZIP2 transporter in plant cells. (**a**,**b**) Confocal laser scanning microscopy images of *N. benthamiana* leaf epidermal cells transiently coexpressing either 35S::eGFP (**a**) or 35S::AsZIP2::eGFP (**b**) with the plasma membrane marker CERK1::DsRed driven by the 35S promoter. Left panel (I): GFP channel; center panel (II): mCherry channel; right panel (III): Merged. Scale bar, 50 μm.

Since the biological functions of proteins are closely related to their subcellular locations in plant cells [110,111], we generated C-terminal-enhanced green fluorescent protein (eGFP) fusion of the AsZIP2 protein driven by the 35S promoter to confirm the AsZIP2 localization in plant cells. We next performed the protein colocalization experiment in the leaf epidermal cells of tobacco using the specific plasma membrane marker [96,111]. As expected, the expression of the plasma membrane marker revealed that the CERK1-DsRed fusion protein was correctly targeted to the plasma membrane of tobacco leaf epidermal cells (Figure 7). Nevertheless, the free eGFP signal was visualized in the plasma membrane, cytoplasm and nucleus of tobacco cells (Figure 7a). Confocal microscopy analysis further confirmed that the AsZIP2-eGFP fusion protein was restrictively co-localized with the plasma membrane marker CERK1-DsRed (Figure 7b). Overall, this result reveals that the AsZIP2 protein is localized at the plasma membrane, implying its potential Zn transport role in *A. sinicus*.

### 3.10. Overexpression of AsZIP2 Results in Increased Zn Concentration in A. sinicus Roots under Pi Starvation or High Zn Condition

**Figure 8:**
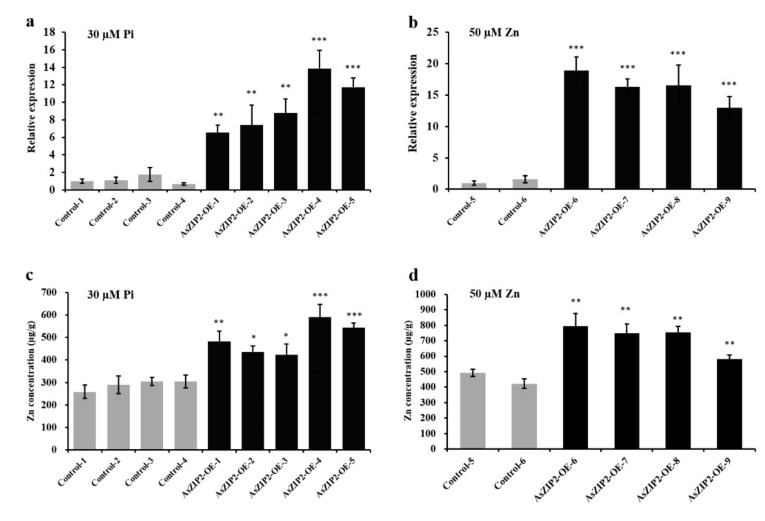
Zinc (Zn) concentration in transgenic *A. sinicus* lines overexpressing *AsZIP2* (*AsZIP2-OE* lines) and in control lines grown under Pi starvation or moderately high Zn supply. (**a**,**b**) Molecular phenotypes in the transgenic *A. sinicus* roots during Pi starvation (30 µM) or moderately high Zn (50 µM ZnCl_2_) conditions. Real time qRT-PCR analysis of *AsZIP2* expression in control and OE lines. *AsZIP2-OE-1* to *AsZIP2-OE-11* represented independent transgenic lines. *AsActin* served as the endogenous control. (**c**) The Zn concentrations in the transgenic roots of 56-d-old *A. sinicus* plants grown in sand cultures exposed to Pi starvation (30 µM). (**d**) The Zn concentrations in the transgenic roots of 56-d-old *A. sinicus* plants grown under moderately high Zn concentration (50 µM ZnCl_2_). Error bars represent the SD for means of three technical replicates. Significant differences between the *AsZIP2-OE* lines and controls: ***, *p*< 0.001; **, *p*< 0.01; *, *p*< 0.05; Student’s *t*-test.

From the above presented results (Figure 6 and Figure 7), we conclude that AsZIP2 behaves as a plasma membrane-localized transporter in Zn transport properties. To next investigate whether the high expression of *AsZIP2* during Pi starvation or excess Zn contributes to the over-accumulation of Zn in roots of *A. sinicus*, we constructed *A. sinicus* transgenic roots overexpressing *AsZIP2* driven by the 35S promoter. The overexpression (OE) of *AsZIP2* in the roots of fourteen transgenic lines was determined by real time qRT-PCR (Figure 8a,b and Figure S7a). The element concentration analysis showed that *A. sinicus* root in all five transgenic lines accumulated Zn levels similar to that of control lines when grown in the standard condition (Figure S7c).

Given the high *AsZIP2* expression in Pi-starved roots or during high Zn supply (see Figure 3 and Figure 5) and the yeast expression results obtained (see Figure 6), we evaluated the Zn concentrations of the *AsZIP2* transgenic lines grown in Pi starvation or excess Zn condition. Under Pi starvation, all five lines, *AsZIP2-OE-1* to *AsZIP2-OE-5*, with high expression of *AsZIP2* (Figure 8a), accumulated much higher level of Zn in roots than did the control lines (Figure 8c). We then examined the Zn levels in the *AsZIP2-OE* lines (Figure 8b) exposed to excess Zn, and similar results were obtained when high supplemental Zn (50 µM) was added to the cultures. As shown in Figure 8d, the Zn concentration was significantly higher in the roots of *AsZIP2-OE-6* to *AsZIP2-OE-9* lines than in control roots.

Collectively, these data reveal that the high expression of *AsZIP2* led to over-accumulation of Zn in *A. sinicus* roots during Pi starvation or Zn excess.

### 3.11. Loss of AsZIP2 Function Leads to Reduced Zn Concentration in Roots of A. sinicus under Low Pi or Excessive Zn Condition

**Figure 9:**
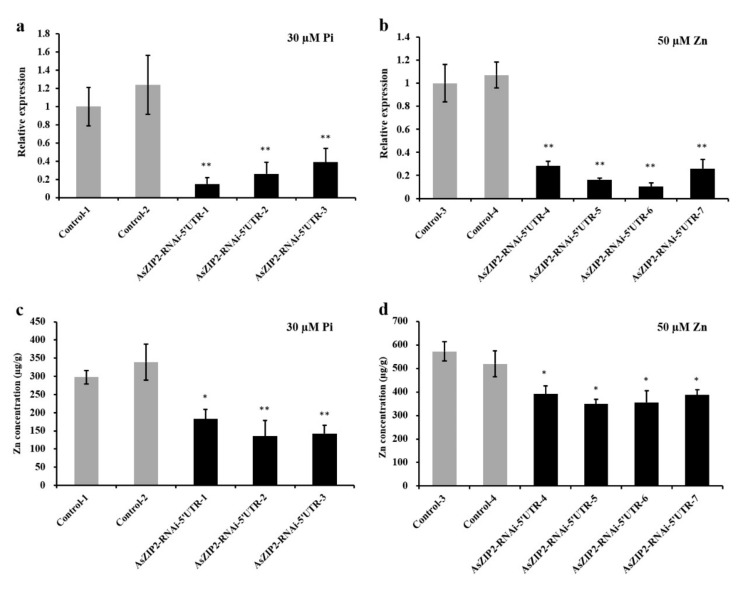
Zn concentration in transgenic *A. sinicus* lines silencing *AsZIP2* (*AsZIP2-RNAi* lines) and in control lines grown under Pi starvation or moderately high Zn supply. (**a**,**b**) Real time qRT-PCR analysis of *AsZIP2* transcript levels in the control and RNAi lines of *A. sinicus* grown under Pi starvation (30 µM) or moderately high Zn (50 µM ZnCl_2_) conditions. *AsZIP2-RNAi-5′UTR-1* to *AsZIP2-RNAi-5′UTR-7* represented independent transgenic lines. *AsActin* served as the control. (**c**) The Zn concentrations in the control and *AsZIP2-RNAi* roots of 56-d-old *A. sinicus* plants grown in cultures exposed to Pi starvation (30 µM). (**d**) The Zn concentrations in the control and *AsZIP2-RNAi* roots of 56-d-old *A. sinicus* plants grown under the moderately high Zn concentration (50 µM ZnCl_2_). Error bars represent the SD for means of three technical replicates. Significant differences between the *AsZIP2-RNAi* lines and controls: **, *p* < 0.01; *, *p* < 0.05; Student’s *t*-test.

The overexpression data gathered above point to a role of the AsZIP2 transporter in root Zn transport when *A. sinicus* plants experience either Pi starvation or excess Zn. To confirm and extend these OE results, we generated the *A. sinicus* hairy root RNAi lines expressing the *AsZIP2-RNAi* constructs driven by the 35S promoter. A total of eleven independent lines noticeably silencing the *AsZIP2* transcript in roots (Figure 9a,b and Figure S7b) were selected for subsequent analysis. We examined whether the silencing of *AsZIP2* affected the Zn uptake in roots of *A. sinicus* by analyzing the Zn concentrations of the *AsZIP2-RNAi* lines. Under standard growth conditions, the root Zn concentrations of the *AsZIP2-RNAi* lines silencing *AsZIP2* transcripts remained unaffected when compared with the control lines (Figure S7d).

We then measured the Zn concentrations in roots of the control and *AsZIP2-RNAi* lines of *A. sinicus* subjected to either Pi starvation or high Zn condition (Figure 9). As expected, the Zn concentrations of three *AsZIP2-RNAi* lines in Pi-starved roots were significantly lower than those of control lines (Figure 9a,c). Similarly, in comparison with control lines, all four *AsZIP2-RNAi* lines exhibited an obviously decreased Zn concentration in roots of *A. sinicus* exposed to excess Zn (Figure 9b,d).

Taken together, these findings confirm that disruption of the *AsZIP2* gene is responsible for the alleviation of excessive Zn accumulation in the *A. sinicus* root during Pi starvation or Zn excess.

## 4. Discussion

As sessile organisms, terrestrial plants are often subjected to various nutrient stresses, such as low Pi and excess Zn concentrations in soil. On one hand, as two fundamentally important fertilizers for maintaining crop yield and quality, Pi and Zn are two of the main factors to sustain plant production. However, the availability of Pi in soil has reduced in many regions due to low pH ferruginous red soils and high Zn fertilized soils. On the other hand, excess Zn materials have been released into soils because of inordinate agro-industrial activities in recent years [53,112,113]. Therefore, crop species experience both Pi starvation and Zn excess stresses simultaneously. Although crosstalk between Pi and Zn nutrients in plants have long been noted in plant nutrition [48,49,50,52,54,55], the effect of Pi starvation on Zn over-accumulation in mycorrhizal plants and molecular basis of this effect are only partially understood. Hence, the investigation of physiological and molecular mechanism of this Pi–Zn crosstalk in plants has both biological and agricultural significance, which is meaningful for plant growth and health. In this study, we demonstrated that Pi deficiency significantly increased the Zn concentrations in roots and shoots of *A. sinicus*, but that Zn over-accumulation was alleviated in *A. sinicus* by high Pi application or AMF inoculation. Our results further reveal that the over-accumulation of Zn in roots results from the up-regulation of a novel ZIP transporter gene *AsZIP2* in *A. sinicus*, which is suppressed by high Pi and mycorrhization.

### 4.1. Pi Starvation-Induced AsZIP2 Is Involved in the Pi–Zn Interaction in A. sinicus

The negative relationship between Pi and Zn nutrients in plants has been extensively studied at the physiological level [48,50,53,56,85], but little is known about the genetic determinants involved in this coordination of Pi and Zn homeostasis in plants. Recently, working models of the crosstalk between Pi and Zn transport and signaling pathways have been proposed in Arabidopsis and rice plants [53,56]. However, the key genes acting as linkers in this Pi and Zn crosstalk remain unclear. In this study, it is noticeable that low levels of Pi triggers Zn over-accumulation in roots and shoots of *A. sinicus* (Figure 1b). Interestingly, the transcription of *AsZIP2* was obviously induced in *A. sinicus* plants under low Pi supply conditions (Figure 3 and Figure 4). This *AsZIP2* expression is positively correlated to the Zn accumulation in Pi-starved *A. sinicus*. It is therefore proposed that this new *AsZIP2* gene encoding protein may contribute to the over-accumulation of Zn concentration in *A. sinicus* plants during Pi deficiency. To test this hypothesis, first, we identified and characterized this *AsZIP2* gene and its protein as a member of the plant ZIP family Zn transporter conserved across dicots and monocots (Figure 2 and Figure S2). Moreover, spatial-temporal expression analysis showed that *AsZIP2* was predominately expressed in the central cylinder of *A. sinicus* roots in a low Pi-dependent manner (Figure 3e,g). This finding indicates that this new AsZIP2 transporter may play a potential role in Zn translocation in roots or roots-to-shoots of *A. sinicus* during Pi starvation, and also raises a new question of whether the expression of *AsZIP2* was activated at the transcriptional level by Pi starvation signaling. As expected, the responsiveness of *AsZIP2* gene to Pi deficiency is consistent with the existence of Pi starvation response *cis*-element P1BS (GNATATNC) in its promoter region (Figure S3), suggesting that *AsZIP2* may be directly induced by Pi starvation through the potential PHR1-P1BS module in *A. sinicus* [108].

In addition, it was found that *AsZIP2* was also up-regulated in roots and shoots of *A. sinicus* by high Zn supply (Figure 3). This result is consistent with previous reports that *M. truncatula MtZIP2* and tobacco *NtZIP11* were highly up-regulated in the roots and shoots by Zn excess [21,45]. In such a context, the induction of *AsZIP2* expression in response to Pi starvation is simply interpreted as a consequence of an increase in plant Zn concentration under such conditions (Figure 1b). Indeed, this interpretation is not correct since the transcript levels of *AsZIP2* in response to Pi starvation might be mediated by PHR1 through its binding to the P1BS *cis*-element presented in the *AsZIP2* promoter (Figure S3), independently of plant Zn nutrition conditions (Figure 1b and Figure 4c,d). Therefore, Pi starvation induced Zn accumulation in *A. sinicus*, possibly resulting from root-to-shoot translocation of Zn by inducing Zn transporter gene *AsZIP2* in the roots. Altogether, this new evidence reveals that AsZIP2 acted in the regulation of Zn transport in *A. sinicus* through Pi starvation signaling.

### 4.2. AsZIP2 Transport Zn in Roots under Pi Starvation or Zn Excess Resulting in Plant Zn Over-Accumulation

AsZIP2 served as a functional Zn transporter in yeast and was localized into the plasma membranes of tobacco epidermal cells. The membrane localization of AsZIP2 is similar to that of the two closely related homologs from *M. truncatula* MtZIP2 [45] and tobacco NtZIP11 [21]. Therefore, it can be concluded that AsZIP2 functions as a Zn transporter involved in the uptake activity of Zn ions from extracellular spaces into the root cells of *A. sinicus*, resulting in high cytoplasmic Zn levels that would disrupt cellular processes. Moreover, overexpression of *AsZIP2* contributed to the over-accumulation of Zn in roots of *A. sinicus* at low Pi or high Zn supply, whereas knock-down of *AsZIP2* resulted in a reduced Zn concentration in roots under such conditions (Figure 8 and Figure 9), but not under standard growth conditions (Figure S7). This was consistent with the very low *AsZIP2* expression levels detected in roots under such standard conditions (Figure 3a,c), suggesting that high expression of *AsZIP2* in roots caused the Zn over-accumulation in *A. sinicus* plants during low Pi or high Zn conditions. To interpret the data showing that changes in *AsZIP2* expression had no significant effect on Zn concentration in roots under standard growth conditions, we propose that AsZIP2 may be functionally redundant with other unidentified members of the ZIP family gene for Zn uptake and homeostasis in *A. sinicus* under such control conditions. In this case, the expression of these unknown *AsZIPs* could compensate for the expression/suppression of *AsZIP2*. Altogether, these findings reveal that AsZIP2 contributes to Zn transport in roots of *A. sinicus* under Pi starvation or Zn excess. However, the underlying mechanism by which AsZIP2 (or its paralogs AsZIP transporters) mediates over-accumulation of Zn in shoots of *A. sinicus* remain largely unknown, and further study is required for a defined mechanism for the involvement of AsZIP2 and/or any new member in *A. sinicus*.

In recent years, it has been well documented that the post-transcriptional and post-translational regulations, such as small RNAs, alternative splicing, proteins interaction, phosphorylation, and ubiquitination, play critical roles in controlling Zn homeostasis by regulating the activities of Zn transporters [114,115,116,117,118,119]; indicating that multiple layers of regulation are involved in plant Zn transport in response to Zn availability. For example, a recent study has uncovered that the plasma membrane-localized IRT1, acting as a transceptor in Arabidopsis, senses the excess of Zn^2+^ in the cytoplasm and recruits the calcium-dependent CBL-interacting protein kinase 23 (CIPK23) to phosphorylate the IRT1 senor, which is ubiquitinated by the E3 ubiquitin ligase IRT1-degradation factor1 (IDF1) and degraded in the vacuole [118]. On a broader scale, the post-transcriptional and post-translational regulations also exist for the nutrient transporters and channels in plants. For example, the boric acid channel NIP5;1 and transporter BOR1 are essential elements for maintaining the boron homeostasis in *A. thaliana*, more importantly, high levels of boron induce downregulation of NIP5;1 and BOR1 through mRNA degradation and protein endocytosis, respectively [120]. Therefore, it would be interesting to investigate the possible post-transcriptional, translational, and post-translational regulations of the AsZIP2 Zn transporter in future studies.

### 4.3. AM Contributes to Plant Tolerance to Zinc by Suppressing AsZIP2 Expression

In spite of Zn being required for plant nutrition, high Zn concentration is toxic for plant growth and disrupts cellular processes [30,53,121]. It is thus important for plant (specially crop) health to manipulate optimal Zn uptake and sustain moderate-high Zn concentration in plants. In this study, our results indicate that high Pi supply reduced the over-accumulation of Zn in both roots and shoots of *A. sinicus* plants (Figure 1b). This finding provided a new approach for nutrient management in crops to alleviate Zn toxicity in the future. Therefore, the manipulation of Pi fertilizer seems to be an effective strategy to decrease the high level of Zn in plants exposed to Zn-contaminated soil [53].

On the other hand, AM *A. sinicus* plants also had a significantly reduced Zn level but much more Pi concentrations than NM plants under the double stresses (low Pi and high Zn). This study again demonstrated that AMF not only facilitate plant nutrition acquisition at low nutrient concentrations in soil, but are also a key element in the phytoremediation of polluted soils [20]. In this context, here, we would like to discuss the question of the contribution of AM to plant tolerance to heavy metal Zn under toxic Zn conditions. Strikingly, the indication that *AsZIP2* expression was repressed in *A. sinicus* roots in response to AM fungal colonization was present in the Pi–Zn interaction and gene expression experiments. This expression pattern was consistent with a previous report that *MtZIP2* was down-regulated in the mycorrhizal roots of *M. truncatula* [45]. It is well known that AM fungal colonization can activate the mycorrhizal Pi uptake pathway to strongly increase *A. sinicus* plant Pi uptake [87,109]. As a result, mycorrhizal *A. sinicus* plants exhibit higher Pi status than NM plants. It is thus proposed that the increased Pi availability driven by AM fungi largely leads to the suppression of *AsZIP2* during AM symbiosis. To confirm this, we examined the effect of different Pi availabilities within AM *A. sinicus* on *AsZIP2* expression. As expected, the physiological and molecular analyses showed that the down-regulation of *AsZIP2* was correlated to increased Pi concentrations in mycorrhizal plants (Figure 5d–f). This suppression pattern of *AsZIP2* in AM roots of *A. sinicus* was consistent with the above results on the NM plants under higher Pi conditions (Figure 3a,b), indicating that AM-driven high Pi status might directly affect *AsZIP2* expression in plants.

However, our results show that the down-regulation of *AsZIP2* was not associated with an increase in Zn concentrations within the mycorrhizal *A. sinicus* at a range from low to moderately high Zn soil concentration (Figure 5h–k). Under this range of soil Zn concentrations, the plant micronutrient Zn uptake was modulated by AM fungus (e.g., *R. irregularis*) [20]. Taken together, the AM-suppressed *AsZIP2* in *A. sinicus* was dependent on the Pi availability but not Zn status, suggesting that the AsZIP2 transporter was strongly suppressed by AM symbiosis in a Pi-dependent manner, resulting in an alleviation in plant Zn toxicity.

### 4.4. The Proposed Working Model in Which AsZIP2 Is Inhibited by Pi and AM Symbiosis

**Figure 10:**
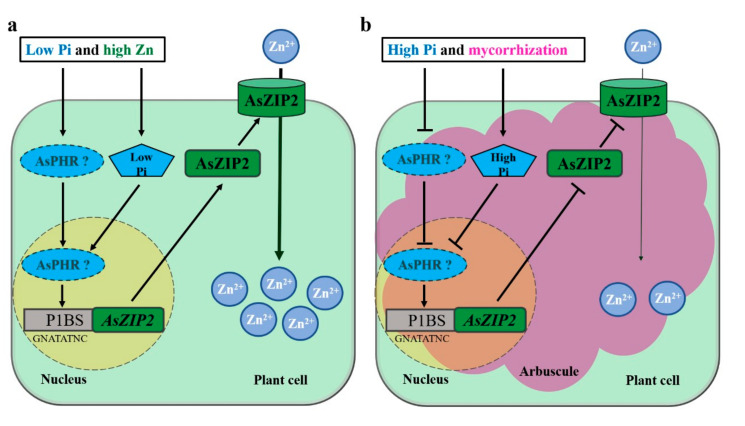
Proposed working model in which the AsZIP2 zinc transporter is repressed by phosphate and arbuscular mycorrhizal symbiosis in *A. sinicus* under moderately high Zn conditions. (**a**) Under Pi starvation, low intracellular Pi availability induces *AsZIP2* expression, possibly dependent on the potential AsPHR-P1BS pathway in *A. sinicus*, due to the presence of P1BS (GNATATNC) motif in the promoter region of *AsZIP2* (see Figure S3); meanwhile, high Zn supply results in low Pi concentration in *A. sinicus* (see Figure 1a), and consequently promotes the transcription of *AsZIP2* to facilitate the activation of Zn transporter AsZIP2 in the plasma membrane. Both the processes lead to the over-accumulation of Zn^2+^ in plant cells. (**b**) Under high Pi conditions, high Pi availability reduces *AsZIP2* expression via the potentially inactivated AsPHR-P1BS module. On the other hand, arbuscular mycorrhization significantly increases cellular Pi concentration, which can also repress the *AsZIP2* expression in a similar manner, leading to less Zn^2+^ accumulation in *A. sinicus*. The solid arrows present the positive influences or interactions, whereas the flat-ended lines indicate the negative influences or interactions; the question marks indicate a non-confirmed AsPHR-P1BS module that possibly controls *AsZIP2* expression in *A. sinicus*.

Based on our findings, we propose a draft model for AsZIP2 acting in the regulation of Zn transport in the roots of *A. sinicus* through Pi starvation signaling (Figure 10). Under Pi starvation, in NM roots (Figure 10a), the low intracellular Pi concentration triggers transcription of *AsZIP2*; an induction that is possibly dependent on the predicted AsPHR-P1BS module in *A. sinicus* because of the existence of Pi starvation response *cis*-element P1BS (GNATATNC) motif in the *AsZIP2* promoter region (Figure S3). On the other hand, a high level of Zn supply (or when present in excess, Zn^2+^ and ZnO NPs-derived Zn^2+^ in soil [80]) induces Pi starvation in plants (Figure 1a) and low Pi concentration in *A. sinicus* resulting from the down-regulation of Pi transporter genes *AsPT2* and *AsPT3* in the roots (Figure 4a,b); this consequently activates the transcription of *AsZIP2* to facilitate the localization of the AsZIP2 Zn transporter in the plasma membrane of the plant cell, suggesting that Zn indirectly regulates the transcript levels of *AsZIP2*. Both cellular processes result in the over-accumulation of Zn^2+^ in root cells. During AM symbiosis (Figure 10b), moderately high Pi availability suppresses *AsZIP2* expression through the potential inactivated AsPHR-P1BS module. On the other hand, arbuscular mycorrhization significantly increases cellular Pi concentration, which might also repress *AsZIP2* expression in a similar manner, resulting in much less accumulation of Zn^2+^ concentration in mycorrhizal roots of *A. sinicus*. Therefore, this study shows that AM contributes to the Zn tolerance of *A. sinicus* through the suppression of *AsZIP2* expression, suggesting that the manipulation of AM fungi may be a friendly, sustainable way to decrease the over-accumulation of excessive Zn in crop species that have been exposed to Zn- or ZnO NPs-contaminated soil.

## 5. Conclusions and Future Perspectives

In conclusion, the present study showed the physiological and molecular mechanism of Pi-Zn interaction in *A. sinicus* under NM and AM conditions. Interestingly, low levels of Pi significantly increased the Zn concentration in *A. sinicus*, associated with the high expression of *AsZIP2* in *A. sinicus*. Importantly, we confirmed that this conserved AsZIP2, belonging to the plant ZIP family, was induced in roots by Pi starvation and Zn excess, and its protein was localized to the plasma membrane of plant cells and served as a Zn transporter. Moreover, the AsZIP2 transporter contributed to the over-accumulation of Zn concentration in roots during low Pi or high Zn supply, but was suppressed by AM fungal colonization in a Pi-dependent manner. Therefore, our findings reveal that the AsZIP2 transporter, a member of the plant ZIP2 subfamily, exerted adverse effects on plants against Zn excess under high Zn conditions in soil, and also illustrated a defined molecular mechanism by which low levels of Pi trigger Zn over-accumulation in roots by increasing *AsZIP2* expression. More importantly, these findings provide new insights into the way arbuscular mycorrhizas alleviate over-accumulation of Zn in plants by inhibiting AsZIP2-mediated direct uptake pathways but increasing Pi levels in mycorrhizal plants. In future studies, the members of ZIP2 transporter subfamily and P1BS motifs in their promoters will be used for loss-of-function by gene editing in crops to reduce the excessive Zn uptake. These new findings provide new avenues for nutrient management or AM fungi inoculation and genetic modification in crops to alleviate Zn toxicity in future.

## Data Availability

All data analyzed in this study are included in this article and its supplementary information files.

## Supplementary Materials

The following are available online at https://www.mdpi.com/article/10.3390/jof7110892/s1, Figure S1. Effect of the external Pi and Zn availabilities on the arbuscular mycorrhizal symbiosis in *A. sinicus*. Figure S2. Gene structure and topology analysis of *A. sinicus AsZIP2*. Figure S3. The P1BS-motif is present in the promoters of the *AsZIP2* from *A. sinicus* as well as the ZIP2 subfamily genes from other plant species. Figure S4. Transcription of the arbuscular mycorrhiza-specific *AsPT4* in mycorrhizal roots of *A. sinicus* in response to Pi and Zn availabilities. Figure S5. Transcription profiles of *AsZIP2* in the roots and shoots of *A. sinicus*. Figure S6. Effect of external Zn concentrations on AM symbiosis in *A. sinicus* roots. Figure S7. Effect of *AsZIP2*-overexpression (*AsZIP2-OE*) and *AsZIP2*-silencing (*AsZIP2-RNAi*) on Zn concentration in roots of *A. sinicus* under standard growth conditions. Table S1. A list of the primers used in this study. Table S2. The accession numbers of the plant ZIP family proteins used in this study.
