# Supplementary material for: Phosphorus Starvation- and Zinc Excess-Induced Astragalus sinicus AsZIP2 Zinc Transporter Is Suppressed by Arbuscular Mycorrhizal Symbiosis
Source: J Fungi (Basel). 2021 Oct 22;7(11):892. doi: 10.3390/jof7110892 (PMC8623892; doi:10.3390/jof7110892)
Supplement: Supplementary file 1 [file jof-07-00892-s001.zip › jof-1414587-supplementary.pdf]

Supporting Information

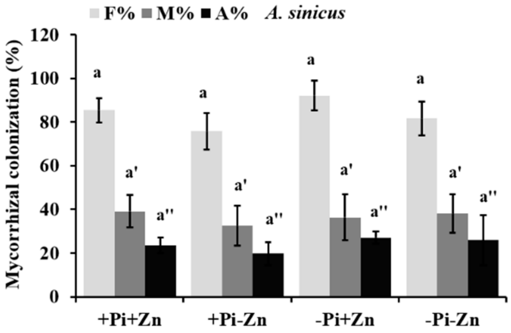

Figure S1. Effect of the external Pi and Zn availabilities on the arbuscular mycorrhizal symbiosis in *A. sinicus*. *A. sinicus* were grown in pot cultures treated with 300 μM Pi and 50 μM Zn (+Pi+Zn), 300 μM Pi and 0.5 μM Zn (+Pi-Zn), 30 μM Pi and 50 μM Zn (-Pi+Zn) or 30 μM Pi and 0.5 μM Zn (-Pi-Zn). 14-d-old *A. sinicus* seedling roots were colonized by *R. irregularis* at 42 dpi, and colonization level was quantified in roots of 56-d-old mycorrhizal plants after WGA488 staining. F%, the total colonization frequency; M%, the percentage of mycorrhizal intensity; A%, the percentage of arbuscule abundance. Error bars represent the SD for means of three biological replicates. The same letters indicate non-significant differences at $P < 0.05$, based on the Duncan's multiple range test.

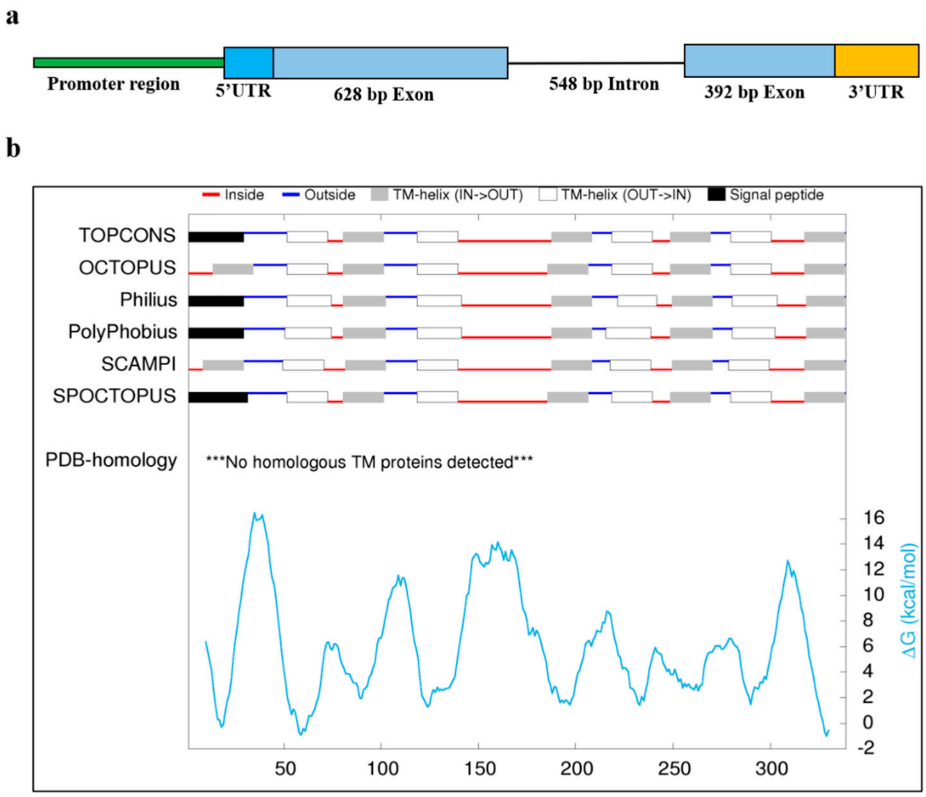

Figure S2. Gene structure and topology analysis of *A. sinicus AsZIP2*. (a) The *AsZIP2* gene corresponds to MZ636517 and consists of two exons (light blue boxes). The dark blue and orange boxes are represented 5′UTR and 3′UTR regions of *AsZIP2*, respectively. The green line upstream of the 5′UTR represents the promoter region of *AsZIP2*. (b) The encoded membrane protein AsZIP2 from *A. sinicus* comprises 339 amino acids and 8 transmembrane domains (TM-helixes). The TOPCONS program (http://topcons.cbr.su.se/) predicts that AsZIP2 (QYE52148) is a membrane intrinsic protein containing 8 TM domains.

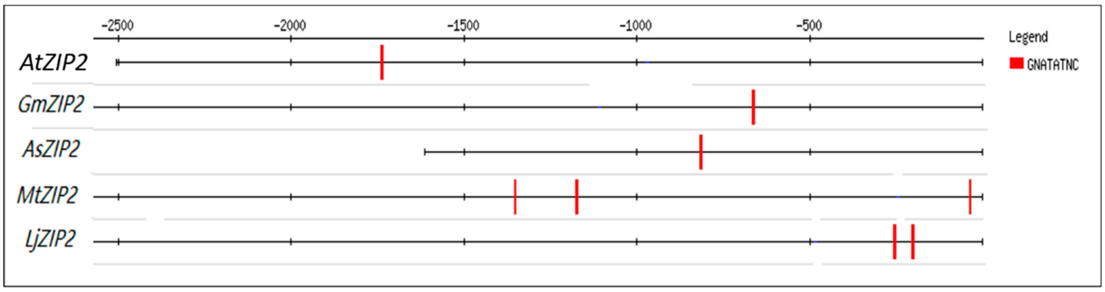

Figure S3. The P1BS-motif is present in the promoters of the *AsZIP2* from *A. sinicus* as well as the ZIP2 subfamily genes from other plant species. The P1BS-motif (GNATATNC), which is the binging site of the PHR1 and PHR1-like transcription factors in plants, was screened in the promoter regions of the plant ZIP2 subfamily genes by the DNA-pattern matching analysis (http://rsat.ulb. ac.be/rsat/). The GNATATNC motif is found in the promoter regions of *AtZIP2* (in *Arabidopsis thaliana*), *GmZIP2* (in *Glycine max*), *AsZIP2* (in *A. sinicus*), *MtZIP2* (in *Medicago truncatula*), and *LjZIP2* (in *Lotus japonicus*), whereas these promoter sequences do not contain the RTGTCGACAY motif, which is directly activated by the bZIP19/23 transcription factors during Zn starvation.

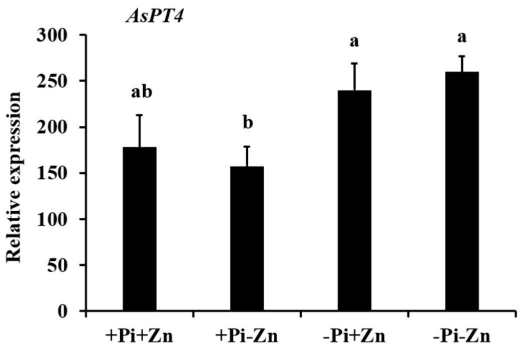

Figure S4. Transcription of the arbuscular mycorrhiza-specific *AsPT4* in mycorrhizal roots of *A. sinicus* in response to Pi and Zn availabilities. The 56-d-old *A. sinicus* roots with *R. irregularis* were harvested and transcription levels of *AsPT4* was estimated by real-time qRT-PCR. *AsActin* for *A. sinicus* was used as the house-keeping gene for normalization. Error bars represents SD from three biological replicates. The different letters indicate statistically significant differences among treatments at $P < 0.05$, based on Duncan's multiple range test.

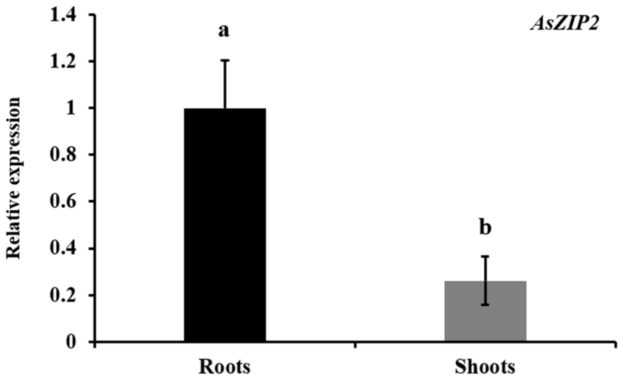

Figure S5. Transcription profiles of *AsZIP2* in the roots and shoots of *A. sinicus*. *A. sinicus* plants were grown under standard (300 μM Pi and 1 μM Zn) conditions. 56-d-old roots and shoots of *A. sinicus* plants were collected separately and transcript levels of *AsZIP2* was quantified by real-time qRT-PCR. The *AsActin* gene for *A. sinicus* was used as the internal control. Error bars mean standard deviation from three biological replicates. The different letters are statistically significant differences between roots and shoots at $P < 0.05$, based on Student's t-test.

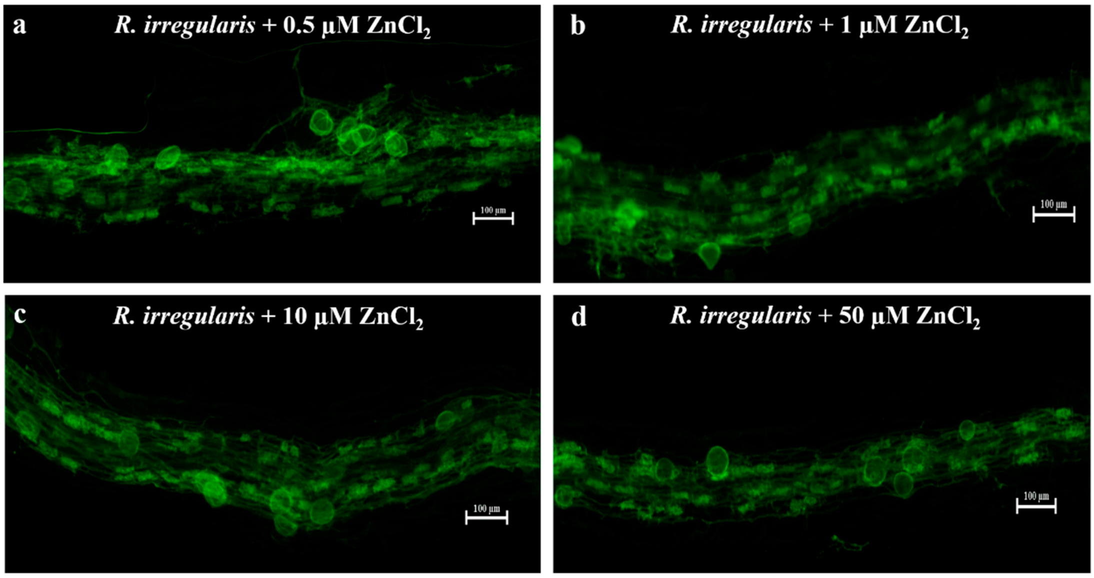

Figure S6. Effect of the external Zn concentrations on the AM symbiosis in *A. sinicus* roots. (a-d) The 56-day-old *A. sinicus* plants were grown in cultures supplied with 0.5, 1, 10, or 50 µM $ZnCl_2$ concentrations, and these *A. sinicus* roots were colonized by *R. irregularis*. Fluorescence images of *R. irregularis* within roots of *A. sinicus* exposed to different Zn concentrations as indicated above. AM roots were stained with WGA488. Scale bars, 100 µm.

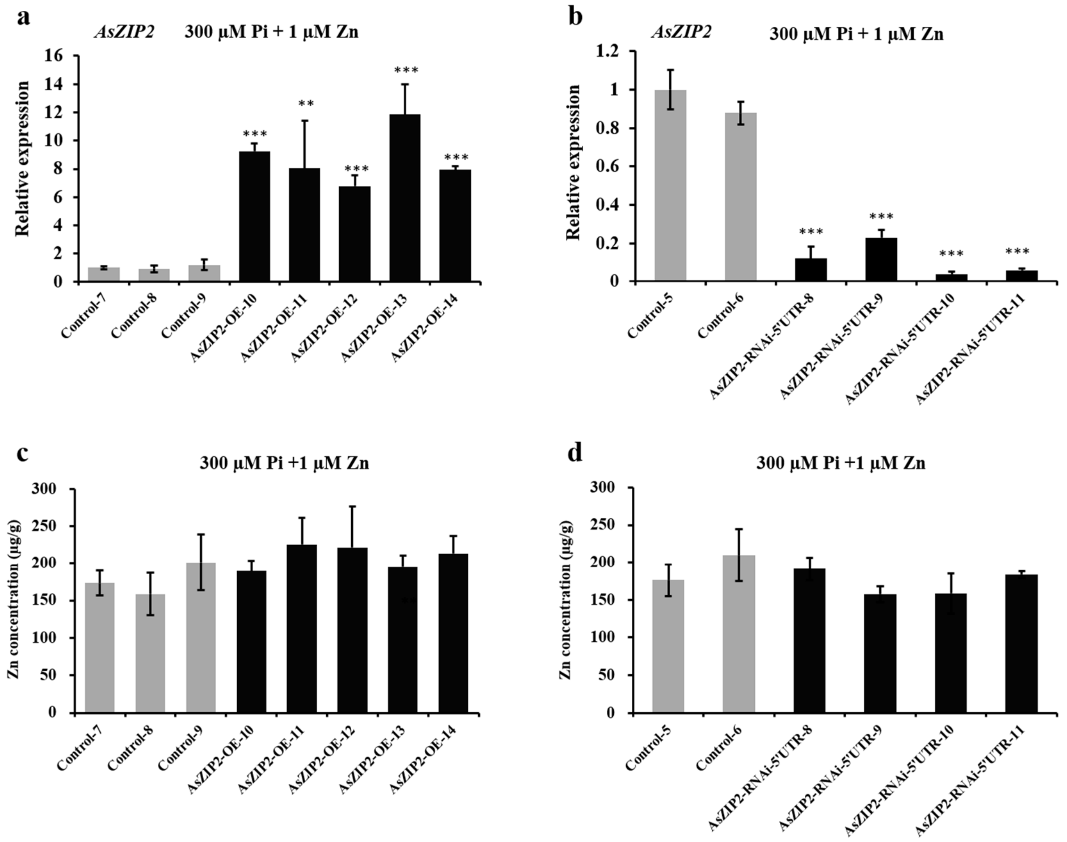

Figure S7. Effect of the *AsZIP2*-overexpression (*AsZIP2-OE*) and *AsZIP2*-silencing (*AsZIP2-RNAi*) on the Zn concentration in roots of *A. sinicus* under standard growth conditions. (a and b) Molecular phenotypes in the transgenic *A. sinicus* roots during standard growth (300 μM Pi and 1 μM $ZnCl_2$) conditions. Real time qRT-PCR analysis of *AsZIP2* expression in the *AsZIP2-OE* (a) and *AsZIP2-RNAi* (b) lines compared with the control lines. *AsActin* from *A. sinicus* served as the endogenous control. (c) The Zn concentrations in the control and *AsZIP2-OE* roots of 56-d-old *A. sinicus* plants under standard growth conditions. (d) The Zn concentrations in the control and *AsZIP2-RNAi* roots of 56-d-old *A. sinicus* plants under standard growth conditions. Error bars represented the SD for means of three technical replicates. Significant differences between the *AsZIP2-OE* (or *AsZIP2-RNAi*) and controls: ***, $P< 0.001$; **, $P< 0.01$; Student's t-test.

Table S1. A list of the primers used in this study.

Primer names	Primer sequences (from 5' to 3')	Use for
AsZIPF1	CAAAACCCTAAAGTCAAC	Degenerate primers
AsZIPR1	TCAATCCCAAATCATGACAAC	Degenerate primers
AsZIPF4	GTGACACTATTCTTCTCATCCTTGC	RT-PCR primers
AsZIPR3	CTCCATGCTTCCCCTTTAGTACCTG	RT-PCR primers
AsZIPF4	GTGACACTATTCTTCTCATCCTTGC	3'RACE primers
AsZIPF5	CTTAGGATGTTACCAAAG	3'RACE primers
AsZIPR4	CCACAACTTTATCTTCCCTCTG	TAIL-PCR primers
AsZIPR5	GAAGAAAAGTCTCATTCCAACC	TAIL-PCR primers
AsZIPR6	CTGAATCACCGTGTCCTCCATGACC	TAIL-PCR primers
AsZIPPF	GGGGACAAGTTTGTACAAAAAAGCAGGCTCAAGTCGCGTCTGCATCTAGAGC	Promoter analysis primers
AsZIPPR	GGGGACCACTTTGTACAAGAAAGCTGGGTGAATGTGTGTACGTATTTTTCTCTC	Promoter analysis primers
AsInvF1	GGTGGTGTGTCTCCTTACTTC	Inverse PCR primers
AsInvF2	CTTGAGTGATTCCAATGAAACCT	Inverse PCR primers
AsZIPR7	CTGAATCACCGTGTCCTCCATGACC	Inverse PCR primers
AsZIPR8	TTGGTTTTGGAGGATGAAGCC	Inverse PCR primers
AsZIP2-OE-F	GGGGTACCATGGCTTCATCCTCCAAAACCAAAG	Overexpression primers
AsZIP2-OE-R	CGCGGATCCTCAATCCCAAATCATTACAAC	Overexpression primers
AsZIP2-ORFF1	GGGGACAAGTTTGTACAAAAAAGCAGGCTATGGCTTCATCCTCCAAAACCAAAG	Subcellular localization in tobacco
AsZIP2-ORFR1	GGGGACCACTTTGTACAAGAAAGCTGGGTTATCCCAAATCATTACAAC	
AsZIP2-Ri-5'-F	GGGGACAAGTTTGTACAAAAAAGCAGGCTCTAGTACAAGTAATACAATTGTAC	RNAi primers
AsZIP2-Ri-5'-R	GGGGACCACTTTGTACAAGAAAGCTGGGTCCAAAACCAAAGCCCTAAAGTC	RNAi primers
AD1	ATGGACTCCAGAGCGGCCGC (G/C/A)N(G/C/A)NNNGGAA	TAIL-PCR long adapters
AD2	ATGGACTCCAGAGCGGCCGC (G/C/T)N(G/C/T)NNNGGTT	TAIL-PCR long adapters
AD3	ATGGACTCCAGAGCGGCCGC(G/C/A)(G/C/A)N(G/C/A)NNNCCAA	TAIL-PCR long adapters
AD4	ATGGACTCCAGAGCGGCCGC(G/C/T)(G/A/T)N(G/C/T)NNNCGGT	TAIL-PCR long adapters
AsZIP2-qF	GTGACACTATTCTTCTCATCCTTGC	Real time qRT-PCR
AsZIP2-qR	CTCCATGCTTCCCCTTTAGTACCTG	Real time qRT-PCR
AsPT2-qF	TCCTGCTGCTCTTACCTTCTACTG	Real time qRT-PCR
AsPT2-qR	CCTCACTCAACTCCTGCACCTT	Real time qRT-PCR
AsPT3-qF	CCTTTGGATTCCTCTATGCTC	Real time qRT-PCR
AsPT3-qR	TTTTCCTTTAGATTCAGGCAC	Real time qRT-PCR
AsPT4-qF	CAGAAACAAAGGGAAGATCATTGG	Real time qRT-PCR
AsPT4-qR	CATGTAATTCAGATCCTTCACACTG	Real time qRT-PCR
AsActin-qF	GTTCTTTTCCAGCCTTCTATGA	*A. sinicus* housekeeping gene for qRT-PCR
AsActin-qR	ATGTTTCCGTACAGATCCTTTC	

Table S2. The accession numbers of the plant ZIP family proteins used in this study.

Gene names	Accession Numbers	Species
AsZIP2	QYE52148	*Astragalus sinicus*
MtZIP2	XP_003597387.1	*Medicago truncatula*
LjZIP2	AFK49261.1	*Lotus japonicus*
AtZIP2	NP_200760.1	*Arabidopsis thaliana*
OsZIP1	XP_015633357.1	*Oryza sativa Japonica Group*
OsZIP2	XP_015628224.1	*Oryza sativa Japonica Group*
PtZIP11	XP_002300374.2	*Populus trichocarpa*
PtZIP12	XP_006369999.2	*Populus trichocarpa*
SlZIP2	NP_001234349.1	*Solanum lycopersicum*
AtZIP11	NP_564703.1	*Arabidopsis thaliana*
MtZIP11	XP_013460015.1	*Medicago truncatula*
GmZIP11	XP_003532170.1	*Glycine max*
GmZIP13	XP_006602141.1	*Glycine max*
GmZIP14	XP_003551465.1	*Glycine max*
GmZIP7	XP_003547009.1	*Glycine max*
GmZIP3	NP_001304414.2	*Glycine max*
GmZIP2	XP_003543520.3	*Glycine max*
MtZIP6	XP_003615761.1	*Medicago truncatula*
GmZIP6	XP_003544893.1	*Glycine max*
PtZIP6	XP_002299993.1	*Populus trichocarpa*
SlZIP6	XP_010321288.1	*Solanum lycopersicum*
AtZIP6	NP_180569.1	*Arabidopsis thaliana*
OsZIP6	XP_015640855.1	*Oryza sativa Japonica Group*
AtZIP7	NP_178488.1	*Arabidopsis thaliana*
GmIRT1	XP_003520144.1	*Glycine max*
MtIRT2	XP_024628929.2	*Medicago truncatula*
SlIRT3	XP_019067417.1	*Solanum lycopersicum*
GmZIP10	NP_001240277.1	*Glycine max*
GmZIP12	NP_001274385.1	*Glycine max*
MtIRT1	XP_003607852.1	*Medicago truncatula*
AtZIP8	Q8S3W4.1	*Arabidopsis thaliana*
AtIRT1	NP_567590.3	*Arabidopsis thaliana*
SlIRT2	NP_567590.3	*Solanum lycopersicum*
AtZIP10	NP_174411.2	*Arabidopsis thaliana*
SlIRT1	NP_001234248.1	*Solanum lycopersicum*
OsZIP10	XP_015641360.1	*Oryza sativa Japonica Group*
OsZIP7	XP_015637712.1	*Oryza sativa Japonica Group*
SlZIP4	XP_004245100.1	*Solanum lycopersicum*
AtZIP9	NP_195028.1	*Arabidopsis thaliana*
AtZIP4	NP_001318977.1	*Arabidopsis thaliana*
PtZIP4	XP_002324173.2	*Populus trichocarpa*
MtZIP4	XP_003603149.3	*Medicago truncatula*
GmZIP4	XP_006581291.1	*Glycine max*
GmZIP5	XP_003526000.2	*Glycine max*
MtZIP5	XP_013461166.1	*Medicago truncatula*
MtZIP8	XP_013461171.1	*Medicago truncatula*
MtZIP5.1	XP_003601469.1	*Medicago truncatula*
MtZIP8.1	XP_024635724.1	*Medicago truncatula*
SlZIP5	XP_004243896.1	*Solanum lycopersicum*
SlZIP7	XP_004243649.1	*Solanum lycopersicum*
SlZIP8	XP_004231600.1	*Solanum lycopersicum*
PtZIP8	XP_002307860.3	*Populus trichocarpa*
PtZIP2	XP_002312231.2	*Populus trichocarpa*
PtZIP3	XP_002315075.2	*Populus trichocarpa*
AtZIP1	NP_187881.1	*Arabidopsis thaliana*
MtZIP1	XP_013464193.1	*Medicago truncatula*
GmZIP1	XP_003531480.1	*Glycine max*

GmZIP8	XP_040865466.1	*Glycine max*
GmZIP9	XP_003546826.1	*Glycine max*
OsZIP8	XP_015644611.1	*Oryza sativa Japonica Group*
OsZIP9	XP_015637508.1	*Oryza sativa Japonica Group*
OsZIP5	XP_015637510.1	*Oryza sativa Japonica Group*
OsZIP3	XP_015635611.1	*Oryza sativa Japonica Group*
OsZIP4	XP_015650399.1	*Oryza sativa Japonica Group*
